# Manual and automated tissue segmentation confirm the impact of thalamus atrophy on cognition in multiple sclerosis: A multicenter study

**DOI:** 10.1016/j.nicl.2020.102549

**Published:** 2020-12-25

**Authors:** Jessica Burggraaff, Yao Liu, Juan C. Prieto, Jorge Simoes, Alexandra de Sitter, Serena Ruggieri, Iman Brouwer, Birgit I. Lissenberg-Witte, Mara A. Rocca, Paola Valsasina, Stefan Ropele, Claudio Gasperini, Antonio Gallo, Deborah Pareto, Jaume Sastre-Garriga, Christian Enzinger, Massimo Filippi, Nicola De Stefano, Olga Ciccarelli, Hanneke E. Hulst, Mike P. Wattjes, Frederik Barkhof, Bernard M.J. Uitdehaag, Hugo Vrenken, Charles R.G. Guttmann

**Affiliations:** a Department of Neurology, MS Center Amsterdam, Amsterdam Neuroscience, Amsterdam UMC, Location VUmc, De Boelelaan 1117, 1118, 1081 HV Amsterdam, The Netherlands; b Department of Radiology and Nuclear Medicine, MS Center Amsterdam, Amsterdam Neuroscience, Amsterdam UMC, Location VUmc, De Boelelaan 1117, 1118, 1081 HV Amsterdam, The Netherlands; c Center for Neurological Imaging, Department of Radiology, Brigham and Women’s Hospital, Harvard Medical School, 1249 Boylston Street, Boston, MA 02215, USA; d Department of Human Neurosciences, “Sapienza” University of Rome, Piazzale Aldo Moro, 5, 00185 Roma RM, Italy; e Department of Neurosciences, San Camillo Forlanini Hospital, Circonvallazione Gianicolense, 87, 00152 Roma RM, Italy; f Department of Epidemiology and Biostatistics, Amsterdam UMC, Location VUmc, De Boelelaan 1089a, 1081 HV Amsterdam, the Netherlands; g Neuroimaging Research Unit, Institute of Experimental Neurology, Division of Neuroscience, and Neurology Unit, San Raffaele Scientific Institute, Via Olgettina, 58, 20132 Milano MI, Italy; h Neurology Unit, San Raffaele Scientific Institute, Via Olgettina, 58, 20132 Milano MI, Italy; i Department of Neurology, Medical University of Graz, Auenbruggerplatz 22, 8036 Graz, Austria; j Division of Neurology and 3T MRI Research Center, Department of Advanced Medical and Surgical Sciences, University of Campania “Luigi Vanvitelli”, Viale Abramo Lincoln, 5, 81100 Caserta, CE, Napoli, Italy; k Section of Neuroradiology and MRI Unit, Department of Radiology, University Hospital iValld’Hebron, Autonomous University of Barcelona, Passeig de la Vall d'Hebron 119-129, 08035 Barcelona, Spain; l Department of Neurology, University Hospital iValld’Hebron, Autonomous University of Barcelona, Passeig de la Vall d'Hebron 119-129, 08035 Barcelona, Spain; m Division of Neuroradiology, Vascular and Interventional Radiology, Department of Radiology, Medical University of Graz, Auenbruggerplatz 22, 8036 Graz, Austria; n Neurophysiology Unit, San Raffaele Scientific Institute, and ^14^Vita-Salute San Raffaele University, Via Olgettina, 58, 20132 Milano, MI, Italy; o Department of Neurological and Behavioural Sciences, University of Siena, 53100 Siena SI, Italy; p Department of Neuroinflammation UCL, Queen Square Institute of Neurology UCL, Queen Square, London WC1N 3BG, United Kingdom; q Department of Anatomy and Neurosciences, Amsterdam Neuroscience, MS Center Amsterdam, Amsterdam UMC, Vrije Universiteit Amsterdam, De Boelelaan 1108, P.O. Box 7057, 1007 MB, Amsterdam, The Netherlands; r Department of Diagnostic and Interventional Neuroradiology, Hannover Medical School, Hannover, Carl-Neuberg-Straße, 30625 Hannover, Germany; s Institutes of Neurology & Healthcare Engineering, UCL, 235 Euston Rd, Bloomsbury, London NW1 2BU, United Kingdom

**Keywords:** Multiple Sclerosis, MRI, Cognition, Thalamus, Deep grey matter, Atrophy, Segmentation, BRB-N, Brief Repeatable Battery of Neuropsychological Tests, CAT12, Computational Anatomy Toolbox for Statistical Parametric Mapping 12, CI, cognitively impaired and preserved (CP), CII, cognitive impairment index, CNR, contrast-to-noise ratio, CP, cognitively preserved, EDSS, Expanded Disability Status Scale, eTIV, estimated total intracranial volume, FSL-FIRST, FMRIB Integrated Registration and Segmentation Tool, GIF, Geodesic Information Flows, GM, grey matter, GMV, grey matter volume, ICC, intraclass correlation coefficient, MS, Multiple Sclerosis, NBV, Normalized brain volume, NGMV, Normalized grey matter volume, NWMV, Normalized white matter volume, IPS, information processing speed, HC, healthy control, PASAT, Paced Auditory Serial Addition Test, RRMS, Relapsing-Remitting Multiple Sclerosis, SD, standard deviations, SDMT, Symbol Digit Modalities Test, SPM12, Statistical Parametric Mapping 12, SRT, Selective Reminding Test, 10/36 SRT, 10/36 Spatial Recall Test, WCST, Wisconsin Card Sorting Test, WLG, Word List Generation, WM, white matter, WMV, white matter volume, VolBrain, MRI Brain Volumetry System

## Abstract

**Background and rationale:**

Thalamus atrophy has been linked to cognitive decline in multiple sclerosis (MS) using various segmentation methods. We investigated the consistency of the association between thalamus volume and cognition in MS for two common automated segmentation approaches, as well as fully manual outlining.

**Methods:**

Standardized neuropsychological assessment and 3-Tesla 3D-T1-weighted brain MRI were collected (multi-center) from 57 MS patients and 17 healthy controls. Thalamus segmentations were generated manually and using five automated methods. Agreement between the algorithms and manual outlines was assessed with Bland-Altman plots; linear regression assessed the presence of proportional bias. The effect of segmentation method on the separation of cognitively impaired (CI) and preserved (CP) patients was investigated through Generalized Estimating Equations; associations with cognitive measures were investigated using linear mixed models, for each method and vendor.

**Results:**

In smaller thalami, automated methods systematically overestimated volumes compared to manual segmentations [*ρ*=(-0.42)-(-0.76); *p-*values < 0.001). All methods significantly distinguished CI from CP MS patients, except manual outlines of the left thalamus (*p* = 0.23). Poorer global neuropsychological test performance was significantly associated with smaller thalamus volumes bilaterally using all methods. Vendor significantly affected the findings.

**Conclusion:**

Automated and manual thalamus segmentation consistently demonstrated an association between thalamus atrophy and cognitive impairment in MS. However, a proportional bias in smaller thalami and choice of MRI acquisition system might impact the effect size of these findings.

## Highlights

• Thalamus atrophy is associated with cognitive impairment in multiple sclerosis.
• This was confirmed by automated and manual segmentations, but effect sizes varied.
• The algorithms work in a multi-center setting.
• Automated techniques exhibit proportional bias with respect to thalamus size.
• Differences between vendors can affect the robustness of these associations.

## 1 Introduction

Cognitive deficits are present in up to 70% of patients with multiple sclerosis (MS) and have a significant effect on their activities of daily living and quality of life (Amato et al., 2010, Chiaravalloti and DeLuca, 2008, Rao et al., 1991). Disturbances in the domains of attention, information processing speed (IPS), memory and executive skills are major features of the MS cognitive profile and can often be detected already early in the disease course (Amato et al., 2010, Rao et al., 1991, Rogers and Panegyres, 2007).

In MS patients, there is increasing evidence of the relationship between cognitive dysfunction and damage to deep grey matter (GM) structures, which is typically measured in vivo from structural magnetic resonance imaging (MRI) (Amiri et al., 2018, Geurts et al., 2012). Especially thalamus atrophy seems strongly associated with cognitive decline (Filippi et al., 2014, Houtchens et al., 2007, Minagar et al., 2013, Schoonheim et al., 2015, Schoonheim et al., 2012). Therefore, thalamus volume is a potential surrogate outcome measure for cognition in multicenter observational and treatment studies. However, when using different segmentation approaches a considerable amount of variability is found in the measurement of thalamus volume, leading to inconclusive results regarding the correlation with cognitive tests (Amiri et al., 2018, Derakhshan et al., 2010, Houtchens et al., 2007, Popescu et al., 2016).

Currently, several software packages are available for measurement of thalamus volume, most of which employ an atlas-based segmentation approach based on information from healthy control (HC) images (Amiri et al., 2018, Geurts et al., 2012). These have been widely applied in MS, but their accuracy and consistency are impacted by various sources of error related to technical factors (e.g. variations in image intensity and tissue contrast due to different MRI hardware and acquisition parameters), variability due to disease related changes (white matter lesion, parenchymal atrophy, etc.) and other physiological / pathological factors (e.g., age, sex, hydration, vascular risk factors etc.) (Amiri et al., 2018, de Sitter et al., 2020, Gelineau-Morel et al., 2012, Rocca et al., 2017a, Rocca et al., 2017b, Sastre-Garriga et al., 2020). Given the previously reported limitations of image analysis methods, it is important to understand how consistent and reliable the association between thalamus atrophy and cognition is when using different segmentation approaches in MS patients.

Therefore, the primary aim of this study was to assess the replicability and consistency of the association between thalamus volume and cognitive scores for five automated segmentation methods and fully manual outlining, in a large multi-center cohort of relapsing-remitting MS (RRMS) patients. We chose to compare software packages that are well established, freely available, and widely used throughout the neuroimaging MS research community in order to ensure that our findings would be relevant for future MS neuroimaging studies.

## 2 Materials and methods

This study was approved by the Local Ethical Committees on human studies in each participating center and all subjects gave written informed consent prior to study participation.

### 2.1 Subjects

Subjects were recruited from January 2009 to May 2012 as part of a project on imaging correlates of cognitive impairment in MS at 7 European centers (Bisecco et al., 2015, Damjanovic et al., 2017, Preziosa et al., 2016, Rocca et al., 2014, Tillema et al., 2016). Patients had to have a diagnosis of RRMS (Lublin et al., 2014, Polman et al., 2011), no relapse or corticosteroids treatment within the month before scanning and no history of psychiatric conditions, including major depression. Further inclusion criteria for this study required all subjects to be right-handed and aged between 20 and 65 years.

Since manually delineating the thalamus is labor-intensive and time-consuming, a subset of the full multicenter dataset was selected for automated and manual tissue segmentation of the thalamus. A random sample of patients and HCs was selected by H.V., matched on age and sex, using a computer-generated list of random numbers. The final dataset included 57 RRMS patients [37 females; age 38.9 ± 8.5 (mean ± standard deviations (SD) years); 13.0 (7.0–20.0) (median (range)) years of education] and 17 HCs [12 females; age 40.5 ± 6.6 (mean ± SD) years; 17.0 (8.0–20.0) (median (range)) years of education]. See Table 1 for demographic and clinical variables. Patients had a median (range) disease duration of 6.0 (2.0–33.0) years, and a median (range) Expanded Disability Status Scale (EDSS) score of 2.0 (0.0–6.0). Age, sex and education did not differ between HCs and MS patients (*p* = 0.47; *p* = 0.66 and *p* = 0.12, respectively).

**Table 1:** An overview of the cognitive domains and neuropsychological tests.

Cognitive domains	Cognitive tests
Verbal memory	Selective Reminding Test (SRT)
Visuospatial memory	10/36 Spatial Recall Test (10/36 SRT)
Attention / information processing speed	Symbol Digit Modalities Test (SDMT) & Paced Auditory Serial Addition Test (PASAT) 2 and 3 s
Verbal fluency	Word List Generation (WLG)
Executive functions	Wisconsin Card Sorting Test (WCST)

### 2.2 Clinical and cognitive evaluation

Within 48 hours of the MRI acquisition, MS patients underwent a neurological evaluation including EDSS score and a neuropsychological assessment (see table 1), performed at each participating site by experienced neurologists and neuropsychologists, unaware of the MRI results, using validated translations of the neuropsychological tests. For all patients, cognitive performance was assessed by using the Brief Repeatable Battery of Neuropsychological Tests (BRB-N) (Rao et al., 1990), which includes the Selective Reminding Test (SRT) to assess verbal memory; the 10/36 Spatial Recall Test (10/36 SRT) to assess visuospatial memory; the Symbol Digit Modalities Test (SDMT) and Paced Auditory Serial Addition Test (PASAT) 2 and 3 s to assess attention/information processing speed; and the Word List Generation (WLG) test to assess verbal fluency. In addition, the Wisconsin Card Sorting Test (WCST) was administered to evaluate executive function (Heaton et al., 1993). Performance on the WCST was evaluated by computing scores related to the total errors, the number of perseverative errors, and the number of perseverative responses (Heaton et al., 1993).

The Z-scores for each of the domains were calculated (Sepulcre et al., 2006). Patients with at least 2 abnormal test scores [i.e. scores ≤ 2SD from the normative values provided by Boringa et al. for the BRB-N (Boringa et al., 2001) and by Heaton et al. for the WCST (Heaton et al., 1993)] were considered cognitively impaired (CI), as previously described (Damjanovic et al., 2017, Preziosa et al., 2016). In all MS patients, a cognitive impairment index (CII) was determined as an overall measure of cognitive dysfunction for each patient. Briefly, the CII is a continuous variable obtained by a grading system applied to each patient’s score on every cognitive test, dependent on the number of SDs below the mean normative value (Amato et al., 2006, Camp et al., 1999). Hence, the higher the grade, the greater the patient's impairment.

### 2.3 MRI acquisition

MR images were acquired on 3 T scanners (Amsterdam and Naples: Signa, GE Healthcare, Milwaukee, Wisconsin; Barcelona, Graz and London: Magnetom Trio, Siemens, Erlangen, Germany; Milan and Siena: Philips Intera, Best, the Netherlands). The brain imaging sequences included: (a) a dual-echo turbo-spin-echo (TSE) T2-weighted scan: TR = 4000–5380 ms; TE_1_ = 10–23 ms; TE_2_ = 90–102 ms; echo-train length = 5–11; 44 contiguous, 3-mm-thick axial sections parallel to the anterior/posterior commissure plane; matrix = 256 × 192; FOV = 240 × 180 mm2 (rectangular FOV = 75%); (b) three-dimensional (3D) T1-weighted scan: TR = 5.5–8.3 ms (for GE Healthcare/Philips Intera scanners) or 1900–2300 ms (for Siemens scanners); TE = 1.7–3.0 ms; flip angle = 8°–12°; 176–192 sagittal sections with thickness = 1 mm and in-plane resolution = 1 × 1 mm. All scans were visually inspected for quality.

### 2.4 MRI analysis of lesions and global atrophy

The analysis of lesions and global atrophy on structural MRI data was done centrally at the Neuroimaging Research Unit (Milan, Italy) by experienced observers under supervision of a neurologist (M.A.R.) with 20 years of experience, blinded to the subjects’ identity. T2 hyperintense lesion volumes (LV) were measured on dual-echo TSE images in a semi-automated fashion using a local thresholding segmentation technique (Jim 6.0 software; Xinapse Systems, Colchester, UK). Normalized brain (NBV), normalized white matter (WM) and grey matter (GM) volumes were measured on 3D T1-weighted scans using the SIENAX software (http://fsl.fmrib.ox.ac.uk/fsl/fslwiki/SIENA) (Smith et al., 2002), after WM lesion-filling with LEAP (Chard, Jackson, Miller, & Wheeler-Kingshott, 2010), using co-registration of the T2 lesion masks to the 3D T1-weighted scans (Popescu et al., 2014).

### 2.5 Thalamus volume measurements

Manual and automated volumetric analyses of the thalamus were performed on 3D T1-weighted data sets.

#### 2.5.1 Manual delineations

Manual volumetric analysis was performed within the online framework of the SPINE virtual laboratory (https://spinevirtuallab.org/)), developed by the Center for Neurological Imaging at Brigham and Women’s Hospital, which can be used for manual tracing of regions-of-interest on MRI. This web-based program allows visualization of MR images in axial, coronal, and sagittal orientations to facilitate 3D anatomical interpretation. The delineations were performed according to a standardized protocol (see supplementary material for a detailed description of the anatomical definitions and detailed outlining instructions) and the voxel-wise labeling process was completely manual; that is, it involved no thresholding, seed-growing, shape fitting or other automated interference. One expert reader manually delineated the whole thalamus on axial slices, in a slice-by-slice manner. To assess the long-term test–retest reliability, a random subset of thalami for nine MR images (4 HCs and 5 MS patients) were delineated in a separate session more than three months later. The reader was a neurologist (J.B.), with specialized training and experience in the anatomical labeling of deep GM structures on MRI, supervised by a neuroradiologist (F.B. with more than 30 years of experience). The reader was blinded to the subject’s clinical characteristics.

#### 2.5.2 Automated segmentation methods

Five automated segmentation programs were used to measure the volume of the thalamus. FreeSurfer, FMRIB Integrated Registration and Segmentation Tool (FSL-FIRST), Computational Anatomy Toolbox for Statistical Parametric Mapping 12 (SPM12) (CAT12), Geodesic Information Flows (GIF) and MRI Brain Volumetry System (VolBrain), which will be described briefly below. Further details of these methods are available in the documentation provided by the developers. We ran the software without user intervention, since this is the mode of operation that would be used when processing patient data of large cohorts.

FreeSurfer’s (http://surfer.nmr.mgh.harvard.edu/) (Dale et al., 1999, Fischl et al., 1999) volume-based stream is designed to preprocess MRI volumes and label subcortical structures. The stream consists of multiple stages: in brief, the first stage is an affine registration with Talairach space specifically designed to be insensitive to pathology and to maximize the accuracy of the final segmentation. This is followed by an initial tissue classification and correction of the variation in intensity resulting from the B1 bias field. Finally, there is a high-dimensional nonlinear volumetric alignment to the Talairach atlas where the final segmentation takes place. The manual editing steps that are recommended for FreeSurfer to adjust for cortical reconstructions were excluded here, since we are focusing on the subcortical output; FreeSurfer was applied as a fully automated software, without the addition of any manual editing steps.

FIRST (Patenaude, Smith, Kennedy, & Jenkinson, 2011) is a model-based segmentation tool also part of FSL (http://www.fmrib.ox.ac.uk/fsl/first/index.html) (Smith et al., 2002). Subcortical brain segmentation is performed using Bayesian shape and appearance models constructed from a set of 336 manually-labeled T1-weighted MR images. FIRST models the outer surface of each deep GM structure as a mesh, using models derived from the reference images and the local intensity profiles around the mesh. Finally, it assigns each voxel in the image an appropriate structure label, taking into account local variations in both surface and shape, as well as the presence of neighboring structures.

The CAT12 toolbox (the successor of VBM8) is an extension to SPM12 (http://www.fil.ion.ucl.ac.uk/spm/software/spm12/) to provide computational anatomy (Mutsaerts et al., 2020). The algorithm allows local variations in the tissue intensity distributions, making it more robust to the presence of pathology such as WM lesions.

GIF software (part of NifySeg: http://cmictig.cs.ucl.ac.uk/niftyweb/ program.php?p5GIF) uses manually created atlases for segmentation of the input images (http://www.neuromorphometrics.com/) (Klein & Tourville, 2012). GIF captures the local variation in morphology and in standard space locations, and has been recommended in previous studies on (deep) GM atrophy in MS (Eshaghi et al., 2018). With the use of an iterative geodesic minimization algorithm and the manual labels, more accurate segmentations are expected (Cardoso et al., 2015).

The VolBrain fully automated pipeline provides volumetric brain information at different scales (Manjón & Coupé, 2016). The proposed pipeline is based on a library of manually labeled templates to perform the segmentation process, constructed from subjects from different publicly available datasets (normal adults, Alzheimer disease and pediatric datasets), including subcortical structure segmentation as proposed by Coupé et al. 2011 (Coupé et al., 2011).

### 2.6 Normalization

To correct for the influence of head size, thalamus volumes were multiplied by the head-normalization factor derived from SIENAX for all segmentation methods, including the manual tracings. Alternatively, FreeSurfer segmentations were divided by the estimated total intracranial volume (eTIV) from FreeSurfer. The unnormalized data were used for the evaluation of agreement between methods; the normalized data served for the association analyses with cognitive outcomes.

#### 2.6.1 Contrast-to-noise ratio

To assess whether there were different tissue contrasts in the T1-weighted images obtained at different sites in this multi-center study, as well as to assess if this was related to the observed relation with cognitive measures, we quantified the contrast-to-noise ratio (CNR) for each thalamus (left and right separately, in each subject). This was done as follows: The mean signal in the thalamus was calculated by eroding the manual thalamus outline once using a 3x3x3 kernel (to avoid any chance of partial volume effects from WM) and applying this as a mask on the N3-corrected T1-weighted image, and calculating the mean signal intensity in that region. The mean signal intensity in the WM bordering the thalamus was obtained similarly, but in this case the mask was created by first dilating the manual thalamus mask once, using a 3x3x3 kernel (here, to avoid any chance of partial volume effects from thalamus in the WM border mask) and then creating a border region around that expanded thalamus mask by dilating three times using a 3x3x3 kernel. The border region was then masked with the SIENAX WM mask and with the inverse of the lesion mask, to exclude GM, CSF and lesions. This WM border mask was then applied on the N3-corrected T1-weighted image and the mean signal intensity was calculated. Subsequently, the standard deviation of the image noise was approximated by taking the standard deviation of the signal in the ventricular CSF. The FreeSurfer ventricles segmentation, after excluding choroid plexus, was eroded once using a 3x3x3 kernel to avoid partial volume effects, and then applied as a mask on the N3-corrected T1-weighted image, and the standard deviation was calculated. Finally, the CNR for that thalamus was calculated by dividing the absolute difference between the mean thalamus signal intensity and the mean border WM signal intensity, by the standard deviation of the ventricles.

### 2.7 Statistical analysis

All data analysis was done using SPSS for Windows version 22.0 (Armonk, NY: IBM Corp). The normality of each variable’s distribution was assessed using histograms and normality plots. Group differences of the demographical and clinical variables, as well as the volumetric MRI quantities and scanner type were evaluated using independent sample T-tests for normally distributed variables, non-parametric analysis (Mann-Whitney) for non-normally distributed variables, and Chi^2^ for categorical variables. Brain T2 and T1 LV were log-transformed due to their skewed distribution. Mean and standard deviation of CNR values were reported both per site and per vendor / scanner type.

Volumetric agreement of the manually and automatically generated thalamus segmentations was evaluated through the intraclass correlation coefficient (ICC) based on a two-way mixed effects model, where people effects are random and measure effects are fixed (McGraw & Wong, 1996). The absolute agreement (ICC “type A”) and consistency (ICC “type C”) were reported. Further, to describe the agreement between different segmentation methods, Bland-Altman plots were created in which the difference of two paired measurements (A-B) was plotted against the average of the two measurements [(A + B)/2] (Altman and Bland, 1983, Giavarina, 2015). We ran a One-Sample T-Test to examine whether the mean of the difference equals 0, and a linear regression [Pearson rho (*ρ*)] to evaluate whether a proportional bias was present. In the Bland-Altman plot this bias will be reflected in the scatter points with a trend to high or low values of the difference across the range of values of the average.

Intra-rater reliability of the manual delineations was evaluated through the ICC as described above, reporting the absolute agreement. We used Koo’s criteria to interpret the ICCs: values < 0.5 are indicative of ‘poor’ reliability, values between 0.5 and 0.75 indicate ‘moderate’ reliability, values between 0.75 and 0.9 indicate ‘good’ reliability, and values greater than 0.90 indicate ‘excellent’ reliability (Koo & Li, 2016).

The ability of the thalamus volumes to distinguish between CI and CP MS patients was compared between different segmentation methods by using Generalized Estimating Equations with logit link function and an unstructured covariance matrix, corrected for age. Correlations of cognition with thalamus volumes were investigated using linear mixed models CII and cognitive domain Z-scores as the dependent variables, adjusting for age and with random effects for subject and center, comparing the results between the different segmentation methods. Sex and education were not significantly different between CI and CP patients and were not retained in the models. To assess the influence of vendor, we additionally performed the same general linear regression analysis with CII as the dependent variable for each method, per vendor.

A p-value of<0.05 was considered statistically significant. As the main goal of our study was to investigate the replicability of the association between thalamus volume and cognitive scores using different automated segmentation methods, we did not correct for multiple comparisons to address possible type I errors.

## 3 Results

### 3.1 Subject characteristics

Table 2 summarizes the main demographic, clinical and MRI characteristics of the HCs and MS patients, as well as CP and CI MS patient subgroups. Twenty-two (39%) MS patients were classified as CI. Compared with CP, CI patients were older (*p* = 0.01) and had a higher EDSS score (*p* = 0.025); whereas no difference was found for sex (*p* = 0.33), education (*p* = 0.52) and disease duration (*p* = 0.83). As a consequence, age was included as nuisance covariate in the regression models. Compared to HCs, MS patients had lower NBV (*p* = 0.001), NWMV (*p* = 0.01) and NGMV (*p* < 0.05). Except NWMV (*p* = 0.33), all MRI volumes were more altered in CI than in CP patients (all *p*-values < 0.05), including T2 LV (*p* < 0.01). The cognitive domains most frequently affected were attention / IPS (32% of the MS patients), executive function (23%), verbal memory (19%), visuospatial memory (16%) and verbal fluency (16%). The distribution of vendors across the HC and MS patient subgroups was similar (MS vs HC: *p* = 0.08; CI vs CP: *p* = 0.51). Table 3 lists the number of subjects per center and MR scanner type. CNR values by site and hemisphere are also included, displaying some heterogeneity between sites in this multi-center study.

**Table 2:** Demographic, clinical and MRI characteristics of healthy controls and cognitively preserved and impaired patients.

				MS patients		
	HC (n = 17)	MS (n = 57)	p	CP (n = 35)	CI (n = 22)	p
*Demographic Characteristics*						
Age (in years)^a^	40.5 ± 6.6	38.9 ± 8.5	0.47	36.6 ± 8.1	42.5 ± 7.9	**0.010**
Sex (Female / Male)	12 / 5	37 / 20	0.66	21 / 14	16 / 6	0.33
Education (in years)^b^	16.5 (12.0–18.3)	13.0 (12.0–17.0)	0.12	13.0 (13.0–17.0)	13.0 (11.8–17.0)	0.52
*MS Characteristics*						
Disease duration (in years)^b^		6.0 (4.0–10.0)	–	6.3 (4.0–10.0)	6.0 (4.8–10.7)	0.83
EDSS^b^		2.0 (1.5–2.5)	–	2.0 (1.0–2.0)	2.0 (2.0–4.0)	**0.025**
*MRI Characteristics*						
T2-lesion volume (mL)^b^	–	–		3.16 (1.48 – 6.66)	8.69 (3.02 – 26.24)	**0.001**
NBV (L)^a^	1.53 ± 0.07	1.44 ± 0.11	**0.001**	1.47 ± 1.00	1.40 ± 0.11	**0.012**
NWMV (L)^a^	0.71 ± 0.05	0.66 ± 0.07	**0.013**	0.67 ± 0.06	0.65 ± 0.09	0.33
NGMV (L)^a^	0.82 ± 0.05	0.78 ± 0.07	**0.048**	0.80 ± 0.06	0.75 ± 0.06	**0.003**
*Vendor*						
(GE / Philips / Siemens)	6 / 5 / 6	18 / 18 / 21	0.08	10 / 11 / 14	8 / 7 / 7	0.51

Abbreviations: CI = cognitively impaired; CP = cognitively preserved; EDSS = Expanded Disability Status Scale; : HC = healthy controls; MS = multiple sclerosis; NBV = normalized brain volume; NWMV = normalized white matter volume; NGMV = normalized grey matter volume; ^a^ Data are mean (SD) for normally distributed variables; ^b^ Because of non-normal distribution, median and interquartile range are provided; p-values in bold represent significant values.

**Table 3:** An overview of the subjects for each center (MR scanner).

Institute (scanner type)	HC	CP	CI	Total	CNR left thalamus	CNR right thalamus
Barcelona (Siemens, Trio)	2	8	1	11	1.02 ± 0.30	1.12 ± 0.34
Graz (Siemens, Trio)	1	3	2	6	0.82 ± 0.23	1.02 ± 0.23
London (Siemens, Trio)	3	3	4	10	1.18 ± 0.40	1.34 ± 0.43
Amsterdam (GE, Signa HDxt)	3	4	3	10	1.80 ± 0.64	1.79 ± 0.61
Naples (GE, Signa HDxt)	3	6	5	14	2.33 ± 0.60	2.34 ± 0.58
Siena (Philips, Intera)	2	8	1	11	2.03 ± 0.25	2.07 ± 0.26
Milan (Philips, Intera)	3	3	6	12	1.47 ± 0.49	1.52 ± 0.48
Total	17	35	22	74	1.60 ± 0.68	1.67 ± 0.63

Abbreviations CI = cognitively impaired; CNR = contrast-to-noise ratio; CP = cognitively preserved; HC = healthy controls.

### 3.2 Analysis of volumetric agreement

#### 3.2.1 Intraclass correlation analysis

**Fig. 1:**
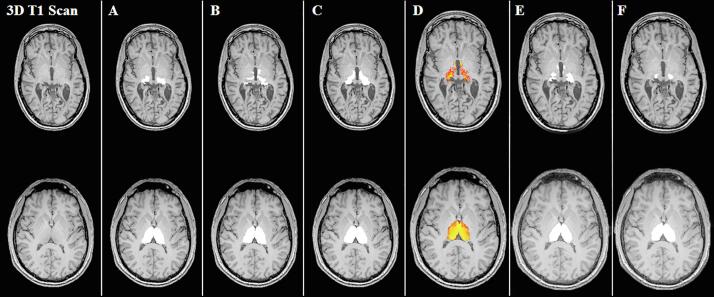
**3D T1-weighted images and thalamus segmentations of manual tracing, FreeSurfer, FSL-FIRST, CAT12, GIF and VolBrain.** Segmentations of the thalamus bilaterally in the axial plane of two MS patients, revealing the inferior portion of the thalamus of one cognitively impaired patient (top row) and the middle part of the thalamus of one cognitively preserved patient (bottom row) for: manual tracings (A), FreeSurfer (B), FSL-FIRST (C), CAT12 (D), GIF (E) and VolBrain (F) segmentations.

Fig. 1 shows examples of the segmentations for each method. In terms of consistency, the agreement between the automatically and manually generated left and right thalamus volumes was good for FreeSurfer and FSL-FIRST, with ICC values ≥ 0.77, and moderate for CAT12, GIF and VolBrain (ICC: 0.61–0.75) (table 4). In terms of absolute agreement, ICC values were good for FreeSurfer (≥0.79), and moderate for FSL-FIRST (≥0.68). Poor absolute agreement was found for left and right thalamus volume measurements from CAT12 (ICC 0.20 and 0.21), GIF (ICC 0.44 and 0.47) and VolBrain (ICC 0.39 and 0.42).

**Table 4:** Intraclass correlation between the absolute (not normalized for head size) thalamus volume measures of different segmentation methods^a,b.^

Intraclass Correlation	Freesurfer – Manual		FSL-FIRST – Manual		CAT12 – Manual		GIF – Manual		VolBrain – Manual	
	Absolute	Consistency	Absolute	Consistency	Absolute	Consistency	Absolute	Consistency	Absolute	Consistency
Left thalamus	0.81	0.80	0.68	0.77	0.20	0.61	0.44	0.60	0.39	0.69
Right thalamus	0.79	0.82	0.69	0.77	0.21	0.64	0.47	0.65	0.42	0.75

Abbreviations: Absolute = absolute agreement; . ^a^ Two-way mixed effects model where people effects are random and measures effects are fixed, single measures. Intraclass correlation coefficients are displayed; ^b^ p= <0.001 for all variables.

#### 3.2.2 Bland-Altman scatter plots and analysis

**Fig. 2:**
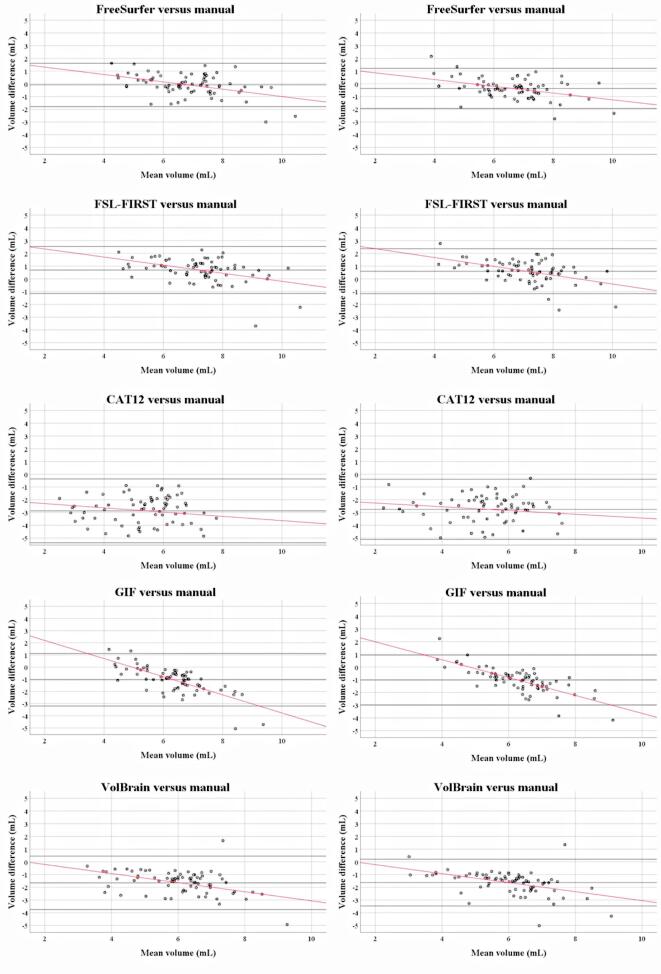
**Bland Altman scatter plots of the unnormalized thalamus volume measurements of the MS patients.** The difference of two paired measurements [(automated–manual) / average] was plotted against the average of the two measurements [(automated + manual) / 2]. Except for CAT12, a proportional bias was observed between the automated and manual thalamus volume measurements, indicated by a trend [linear regression (Pearson rho (*ρ*))] to high and low values of the difference across the range of values of the average.

Fig. 2 and table 5 describe the results of the Bland-Altman scatter plots and analysis of the unnormalized thalamus volume measurements: automated *minus* the manual methods. On average, FreeSurfer left thalamus volumes were similar to the manual output, while right thalami were larger [mean difference (SD): left thalamus: −0.09 (0.85), *p* = 0.39; right thalamus: 0.36 (0.79), *p* < 0.001]. FSL-FIRST obtained larger thalamus volumes for *both* hemispheres [left thalamus: 0.69 (0.92), *p* < 0.001; right thalamus: 0.60 (0.88), *p* < 0.001]. In comparison, the software packages CAT12, GIF and VolBrain obtained smaller thalamus volumes bilaterally (all *p*-values < 0.001). Except for CAT12, a proportional difference with a negative trend was observed in all scatter plots showing the agreement between the automated and manual thalamus volume measurements. In smaller thalami the automated methods appeared to systematically overestimate the thalamus volumes compared to manual outlines, whereas in larger thalami the reverse was found. Qualitatively, the areas with the most disagreement occurred in the superior and inferior parts of the thalami, including the geniculate bodies (see Fig. 1).

**Table 5:** Pairwise Bland-Altman comparisons between segmentation methods.

***Measure***					***Proportional bias***		
	***µ diff***	***SD***	***SE µ***	***p-Value***	***ρ* (rho)^a^**	***t***	***p-Value***
*Freesurfer - Manual*							
Left Thalamus	−0.09	0.85	0.10	0.391	−0.44	−4.14	**<0.001**
Right Thalamus	0.36	0.79	0.09	**<0.001**	−0.42	−3.98	**<0.001**
*FSL-first - Manual*							
Left Thalamus	0.69	0.92	0.11	**<0.001**	−0.44	−4.12	**<0.001**
Right Thalamus	0.60	0.88	0.10	**<0.001**	−0.48	−4.58	**<0.001**
*CAT12 - Manual*							
Left Thalamus	−2.87	1.25	0.15	**<0.001**	−0.17	−1.46	0.15
Right Thalamus	−2.75	1.18	0.14	**<0.001**	−0.16	−1.33	0.19
*GIF - Manual*							
Left Thalamus	−1.04	1.08	0.13	**<0.001**	−0.74	−9.30	**<0.001**
Right Thalamus	−1.02	0.98	0.11	**<0.001**	−0.76	−9.82	**<0.001**
*VolBrain - Manual*							
Left Thalamus	−1.65	1.05	0.12	**<0.001**	−0.42	−3.92	**<0.001**
Right Thalamus	−1.63	0.91	0.11	**<0.001**	−0.47	−4.48	**<0.001**

Abbreviations: µ diff = mean difference; SD = standard deviation; SE µ= standard error of µ; ρ (rho) = Pearson correlation; t = *t*-test statistic; ^a^ Correlation of the volume difference and mean between two measurements; p-value in bold represent significant values.

### 3.3 Reproducibility of manual thalamus outlining

The long-term intra-rater reliability of the manual output, assessed on the images of 9 subjects, was moderate with a median ICC (absolute agreement) of 0.62 (*p* < 0.01) for the left thalamus and 0.63 (*p* < 0.001) for the right thalamus.

### 3.4 Relation of thalamus volume measures with cognition

#### 3.4.1 Thalamus volumes

Table 6 lists the normalized left and right thalamus volumes obtained through manual tracings and automated techniques in CI and CP MS patients. Compared to CP patients, CI patients had smaller thalami based on all methods, excepted the left thalamus volumes obtained through manual outlining (*p* = 0.18) and marginally significant for left thalamus volumes from GIF (*p* = 0.05). All segmentation methods consistently demonstrated smaller thalami in MS patients than in HCs (all *p*-values < 0.001; not shown in the table). In both HCs and MS subjects, the right thalami were smaller than the left thalami for all methods. This difference in left and right thalamus volumes was not statistically significant between methods (*p* = 0.79 for both HCs and MS patients; not shown in the table).

**Table 6:** Normalized thalamus volume measurements and summary of results of the binary logistic regression analysis for cognitively impaired versus cognitively preserved MS patients^a.^

	Thalamic volumes			Binary logistic regression		
	CP (n = 35)	CI (n = 22)	p-Value	OR	95% Conf int. for OR	p-Value
Normalization SIENAX^b^						
*Manual outlines*						
Left thalamus (mL)	8.99 ± 1.37	8.24 ± 2.31	0.18	0.85	0.66 – 1.11	0.23
Right thalamus (mL)	9.06 ± 1.31	7.91 ± 2.18	**0.033**	0.72	0.52 – 0.95	**0.018**
*Freesurfer*						
Left thalamus (mL)	9.02 ± 1.18	8.11 ± 1.44	**0.012**	0.64	0.42 – 0.99	**0.047**
Right thalamus (mL)	8.59 ± 1.07	7.54 ± 1.37	**0.002**	0.53	0.37 – 0.78	**0.001**
*FSL-first*						
Left thalamus (mL)	10.13 ± 0.94	9.05 ± 1.45	**0.004**	0.51	0.40 – 0.66	**<0.001**
Right thalamus (mL)	9.92 ± 0.83	8.90 ± 1.44	**0.005**	0.49	0.35 – 0.68	**<0.001**
*CAT12*						
Left thalamus (mL)	5.34 ± 1.38	4.09 ± 1.93	**0.012**	0.61	0.51 – 0.72	**<0.001**
Right thalamus (mL)	5.38 ± 1.14	4.17 ± 1.95	**0.013**	0.58	0.48 – 0.71	**<0.001**
*GIF*						
Left thalamus (mL)	7.68 ± 0.70	7.17 ± 1.04	0.05	0.55	0.34 – 0.88	**0.013**
Right thalamus (mL)	7.63 ± 0.63	7.08 ± 1.05	**0.033**	0.49	0.31 – 0.79	**0.003**
*VolBrain*						
Left thalamus (mL)	7.02 ± 1.03	5.85 ± 1.48	**0.003**	0.51	0.41 – 0.63	**<0.001**
Right thalamus (mL)	6.99 ± 0.90	5.77 ± 1.44	**0.001**	0.44	0.34 – 0.58	**<0.001**
Fraction of eTIV Freesurfer^c^						
*Freesurfer*						
Left thalamus (10^−3^)	4.71 ± 0.57	4.20 ± 0.63	**0.003**	0.27	0.11 – 0.68	**0.005**
Right thalamus (10^−3^)	4.49 ± 0.55	3.91 ± 0.61	**0.003**	0.17	0.05 – 0.61	**0.006**

Abbreviations CI = cognitively impaired; Conf int = confidence interval; CP = cognitively preserved; OR = odds ratio. ^a^ Data are mean (SD) for normally distributed variables; ^b^ Thalamic volumes were multiplied by the head-normalization factor derived from SIENAX; ^c^ Thalamus volumes were divided by the estimated total intracranial volume (eTIV) from FreeSurfer; p-values in bold represent significant values.

#### 3.4.2 Consistency of discrimination between cognitively impaired and preserved patients

Table 6 summarizes the results of the binary logistic regression analysis for the discrimination between CI and CP MS patients, using the normalized thalamus volume measurements. As expected, a negative effect was found for all segmentation methods, indicating that CI patients were significantly more likely to have smaller thalami than CP patients (odds ratios: 0.44–0.72). No effect was found for manual measurements of the left thalamus (*p* = 0.23). Normalization through FreeSurfer also resulted in negative effects for FreeSurfer segmentations of the left [odds ratio (95% confidence interval): 0.27 (0.11–0.68); *p* = 0.005)] and right thalamus [0.17 (0.05–0.61); *p* = 0.006].

#### 3.4.3 Analysis of correlations with cognition

After normalization through SIENAX, poorer global neuropsychological test performance (higher CII) was significantly associated with lower left and right thalamus volumes using all segmentation methods, (table 7). For example, CII is expected to increase by 1.45 (*p* = 0.021), 1.26 (*p* = 0.002), 1.22 (*p* = 0.002), 1.06 (*p=*<0.001), (1.05 (*p* = 0.013) and 0.65 points (*p* = 0.032), when the left thalamus volume decreases by one centimeter^3^ when obtained through GIF, FreeSurfer, VolBrain, CAT12, FSL-FIRST and manual outlining, respectively. Normalization through FreeSurfer (eTIV) also resulted in significant correlations between CII and thalamus volumes for FreeSurfer. Table 8 shows the associations between CII and thalamus volume measurements for each method, for each scanner vendor (GE, Philips or Siemens) separately. Volumes that were obtained with Siemens scanners resulted in significant correlations for all methods (*p*-values: 0.001–0.031). Philips scans only showed significant correlations when analyzed with CAT12 (bilaterally: *p =* 0.007 and 0.038), FreeSurfer (right thalamus: *p =* 0.045) and FSL-FIRST (left thalamus: *p =* 0.043). No associations were found for any of the methods when applied to GE images. These correlations seem to be in contradiction with the CNR results by vendor, listed at the bottom of Table 8, which show that in fact the CNR values were lowest for Siemens and highest for GE.

**Table 7:** Summary of results of the general linear regression analysis with cognitive scores as the dependent variables^a.^

Thalamic volumes	Cognitive Impairment Index (CII)		Verbal memory		Visual memory		Attention		Fluency		Executive function^b^	
	B (95%CI)	p-Value	B (95%CI)	p-Value	B (95%CI)	p-Value	B (95%CI)	p-Value	B (95%CI)	p-Value	B (95%CI)	p-Value
Normalization SIENAX^c^												
*Manual outlines*												
Left thalamus	−0.65 (-1.23 – (-0.06))	**0.032**	−0.06 (-0.08–0.21)	0.38	0.15 (-0.002–0.30)	0.053	0.16 (0.04–0.28)	**0.010**	0.07 (-0.07–0.22)	0.31	0.19 (-1.44–1.83)	0.81
Right thalamus	−0.72(-1.32 – (-0.13))	**0.017**	0.09 (-0.06–0.23)	0.22	0.17 (0.02–0.32)	**0.032**	0.18 (0.06–0.30)	**0.004**	0.11 (-0.03–0.26)	0.11	−0.08 (-1.74–1.58)	0.92
*Freesurfer*												
Left thalamus	−1.26(-2.05 – (-0.47))	**0.002**	0.17 (-0.03–0.37)	0.091	0.33 (0.12–0.53)	**0.002**	0.32 (0.16–0.47)	**<0.001**	0.18 (-0.02–0.38)	0.076	−0.45 (-2.74–1.85)	0.67
Right thalamus	−1.36(-2.18 – (-0.53))	**0.002**	0.11 (-0.10–0.32)	0.29	0.40 (0.20–0.60)	**<0.001**	0.33 (0.16–0.49)	**<0.001**	0.19 (-0.02–0.40)	0.070	0.63 (-1.76–3.03)	0.60
*FSL-first*												
Left thalamus	−1.05 (-1.87 – (-0.23))	**0.013**	0.09 (-0.12–0.29)	0.40	0.32 (0.12–0.53)	**0.003**	0.31 (0.15–0.47)	**<0.001**	0.15 (-0.06–0.35)	0.16	1.36 (-0.91–3.63)	0.23
Right thalamus	−0.93(-1.81 – (-0.05))	**0.039**	0.05 (-0.17–0.26)	0.67	0.34 (0.12–0.55)	**0.003**	0.30 (0.13–0.47)	**0.001**	0.15 (-0.06–0.37)	0.15	1.22 (-1.18–3.62)	0.31
*CAT12*												
Left thalamus	−1.06 (-1.63 – (-0.49))	**<0.001**	0.07 (-0.08–0.22)	0.34	0.25 (0.10–0.39)	**0.002**	0.26 (0.14–0.37)	**<0.001**	0.13 (-0.01–0.28)	0.072	1.58 (-0.05–3.22)	0.57
Right thalamus	−1.12(-1.71 – (-0.53))	**<0.001**	0.08 (-0.07–0.24)	0.29	0.27 (0.12–0.43)	**0.001**	0.27 (0.16–0.39)	**<0.001**	0.16 (0.004–0.31)	**0.044**	1.74 (-1.74–1.58)	**0.045**
*GIF*												
Left thalamus	−1.45(-2.67 – (-0.23))	**0.021**	0.17 (-0.13–0.47)	0.26	0.40 (0.10–0.71)	**0.010**	0.48 (0.24–0.71)	**<0.001**	0.27 (-0.03–0.57)	0.074	0.26 (-3.16–3.67)	0.88
Right thalamus	−1.36(-2.61 – (-0.11))	**0.033**	0.14 (-0.17–0.44)	0.38	0.39 (0.07–0.70)	**0.016**	0.45 (0.21–0.70)	**<0.001**	0.30 (0.0004–0.60)	0.050	0.40 (-3.06–3.86)	0.82
***VolBrain***												
Left thalamus	−1.22 (-1.98 – (-0.46))	**0.002**	0.12 (-0.07–0.32)	0.20	0.35 (0.16–0.54)	**<0.001**	0.34 (0.19–0.49)	**<0.001**	0.15 (-0.04–0.34)	0.13	1.27 (-0.90–3.45)	0.25
Right thalamus	−1.30(-2.10 – (-0.51))	**0.002**	0.12 (-0.08–0.32)	0.24	0.37 (0.17–0.57)	**<0.001**	0.34 (0.19–0.50)	**<0.001**	0.17 (-0.03–0.37)	0.10	1.63 (-0.62–3.88)	0.15
Fraction of eTIV FreeSurfer^d^												
*FreeSurfer*												
Left thalamus	−2.51(-4.13 – (-0.89))	**0.003**	0.45 (-0.03–0.37)	**0.03**	0.59 (0.16–1.03)	**0.009**	0.65 (0.34–0.96)	**<0.001**	0.10 (-0.32–0.52)	0.63	1.03 (-3.67–5.73)	0.66
Right thalamus	−2.58(-4.24 – (-0.93))	**0.003**	0.30 (-0.12–0.71)	0.16	0.73 (0.32–1.14)	**0.001**	0.65 (0.32–0.97)	**<0.001**	0.11 (-0.32–0.53)	0.62	3.03 (-1.69–7.75)	0.20

Abbreviations: B = unstandardized regression coefficient; CI = confidence interval; ^a^ All regression analysis were corrected for center and age; ^b^ WCST number of perseverative errors; ^c^ Thalamic volumes were multiplied by the head-normalization factor derived from SIENAX; ^d^ Thalamic volumes were divided by the estimated total intracranial volume (eTIV) from FreeSurfer; p-values in bold represent significant values.

**Table 8:** Summary of results of the general linear regression analysis with CII as the dependent variable, for each vendor^a.^

Thalamus volumes^b^	GE (N = 18)		Philips (N = 18)		Siemens (N = 21)	
	B (95%CI)	p-Value	B (95%CI)	p-Value	B (95%CI)	p-Value
*Manual outlines*						
Left thalamus	0.32 (-1.06 – 1.70)	0.63	−0.47 (-1.50 – 0.57)	0.36	−1.32 (-2.02 – (-0.62))	**0.001**
Right thalamus	0.08 (-1.28 – 1.44)	0.90	−0.58 (-1.77 – 0.61)	0.32	−1.15 (-1.85 – (-0.44))	**0.003**
*FreeSurfer*						
Left thalamus	−0.48 (-1.88 – 0.92)	0.48	−1.79 (-3.64 – 0.05)	0.06	−1.48 (-2.46 – (-0.51))	**0.005**
Right thalamus	−0.57 (-2.09 – 0.95)	0.44	−1.65 (-3.26 – (-0.04))	**0.045**	−1.68 (-2.79 – (-0.56))	**0.005**
*FSL-FIRST*						
Left thalamus	−0.05 (-1.40 – 1.30)	0.94	−2.11 (-4.14 – (-0.08))	**0.043**	−1.40 (-2.41 – (-0.39))	**0.009**
Right thalamus	−0.12 (-1.50 – 1.26)	0.86	−1.56 (-3.87 – 0.74)	0.17	−1.34 (-2.45 – (-0.24))	**0.020**
*CAT12*						
Left thalamus	−0.27 (-1.20 – 0.67)	0.56	−2.00 (-3.38 – (-0.62))	**0.007**	−1.38 (-2.17 – (-0.60))	**0.001**
Right thalamus	−0.34 (-1.33 – 0.66)	0.49	−1.51 (-2.94 – (-0.09))	**0.038**	−1.53 (-2.33 – (-0.73))	**0.001**
*GIF*						
Left thalamus	−0.82 (-2.80 – 1.15)	0.39	-0.253 (-6.69 – 1.62)	0.22	−1.62 (-3.08 – (-0.17))	**0.031**
Right thalamus	−0.90 (-2.87 – 1.07)	0.35	−1.48 (-4.91 – 1.94)	0.37	−1.71 (-3.22 – (-0.19))	**0.029**
*VolBrain*						
Left thalamus	−0.22 (-1.54 – 1.10)	0.73	−1.83 (-3.78 – 1.20)	0.06	−1.53 (-2.40 – (-0.65))	**0.002**
Right thalamus	−0.24 (-1.75 – 1.28)	0.75	−1.59 (-3.31 – 0.13)	0.07	−1.65 (-2.57 – (-0.72))	**0.001**
***Contrast-to-noise ratio***	***GE (N = 18)***		***Philips (N = 18)***		***Siemens (N = 21)***	
Left thalamus	2.11 ± 0.66		1.74 ± 0.48		1.04 ± 0.35	
Right thalamus	2.11 ± 0.64		1.78 ± 0.47		1.18 ± 0.37	

Abbreviations: B = unstandardized regression coefficient; CI = confidence interval; CII **=** Cognitive Impairment Index**.**^a^All regression analysis were corrected for center and age; ^b^ Thalamus volumes were multiplied by the head-normalization factor derived from SIENAX; p-values in bold represent significant values.

#### 3.4.4 Analysis of correlations with performance scores on separate cognitive domains

Looking at the correlation with cognitive domain z-scores (table 7), thalamus volume loss was associated with visuospatial memory and attention / IPS based on all methods, excepted a lack of statistically significant association between manually segmented left thalamus volume and visuospatial memory. Based on CAT12, right thalamus volume was associated with verbal fluency (*p* = 0.044) and executive function (*p* = 0.045). No associations were found with the other cognitive domain z-scores. Similar results were found for the normalized (eTIV) FreeSurfer thalamus volume measurements, except that a significant correlation between left thalamus volume loss and verbal memory was also found using this method (*p* = 0.03).

## 4 Discussion

In this multi-center cohort, RRMS patients with relatively mild physical disability and overt CI showed severe thalamus atrophy based on all automated segmentation techniques, as was also evidenced by a unique set of manually defined reference outlines in which the whole thalamus was segmented. Automated and manual tissue segmentation consistently demonstrated a relationship between the degree of thalamus atrophy and cognitive dysfunction, which suggests that the observed association is truly a manifestation of the disease. However, the robustness of these associations was systematically affected by scanner. Somewhat surprisingly, our results showed that images with lower CNR resulted in more significant correlations with cognitive measures, warranting further and more systematic studies of these issues. The differential bias present in smaller and larger thalami should be taken into account when evaluating treatment response of therapeutic interventions.

To our knowledge, this is the first multicenter study that compared automated thalamus segmentation methods and manual outlining, and evaluated their influence on the association of thalamus volume with cognition in MS patients in the presence of MS-related pathologies. Earlier research on this topic considered single-scanner data only (Glaister et al., 2017, Houtchens et al., 2007, Popescu et al., 2016); or compared automated techniques without including manual outlining (Derakhshan et al., 2010, Popescu et al., 2016). When aiming to fully understand the relationship between thalamus atrophy and cognitive decline, automated methods may present a biased picture or reflect spurious correlations, since there have been reports that the algorithms may yield measurement errors that increase with increasing MS pathology such as WM lesions and atrophy (Amiri et al., 2018, Derakhshan et al., 2010, Sastre-Garriga et al., 2020). Taken together, the finding of the present study that expert manual outlining, by and large, resulted in the same associations with cognition as automated methods, is an important confirmation of many earlier reports that have consistently demonstrated more severe thalamus damage in CI patients (Benedict et al., 2013, Houtchens et al., 2007, Minagar et al., 2013, Popescu et al., 2016, Rocca et al., 2018, Schoonheim et al., 2015, Schoonheim et al., 2012). Of note, attention to variations in image characteristics, in particular the CNR between target structure (thalamus) and surrounding tissue, between different scanners and protocols is essential, especially when attempting to minimize the number of patients and observations needed to adequately power clinical trials relying on MRI-derived measurements. Based on our results, which for Siemens showed an unexpected co-occurrence of lowest CNR and significant correlations with cognitive scores across all segmentation software methods, further studies are required to more systematically study the interplay between image contrast, image noise and thalamus segmentation quality.

Similarly to previous studies (Batista et al., 2012, Benedict et al., 2013, Houtchens et al., 2007, Schoonheim et al., 2015, Schoonheim et al., 2012), impaired performance on the domains of attention / IPS and visuospatial memory were associated with thalamus degeneration bilaterally, which was also confirmed through manual outlining. In contrast, we did not find a correlation with executive function, except using CAT12 right thalamus measurements. Impaired IPS is a common and highly invalidating deficit in MS, which can occur at the earliest stages of the disease (Amato et al., 2010, Chiaravalloti and DeLuca, 2008, Rao et al., 1991). With its extensive afferent and efferent interconnections with the midbrain and the cerebral cortex, the thalamus serves as relay station and, thus, thalamus degeneration is likely to contribute to IPS dysfunction (Minagar et al., 2013).

Although the present work confirms that the thalamus is of great clinical relevance to cognitive processes in MS, considerable variations were observed between software packages and scanners, which coincides with the variability reported by previous investigators (Amiri et al., 2018, Glaister et al., 2017, Popescu et al., 2016). In line with an earlier report by Glaister et al, visual inspection of our data showed that the areas with most disagreement occurred in the superior and inferior parts of the thalami, including the geniculate bodies (Glaister et al., 2017). This is probably due to their low contrast compared to surrounding tissue in T1-weighted MRI, which makes it more complicated to trace the edges of the thalamus in these subregions, also manually. The Bland Altman plots revealed that thalamus volumes were on average overestimated by FSL-FIRST and FreeSurfer (excepted left thalamus measurements), while they were systematically underestimated by CAT12, GIF and VolBrain, which is in line with an earlier publication on this topic (de Sitter et al., 2020). It appeared that the absolute agreement for CAT12 (ICC: 0.20–0.21), GIF and VolBrain (ICCs between 0.39 and 0.47) in our study were much worse than previously reported by de Sitter et al. (2020). However, different study populations and combined manual segmentations created by majority voting were used in previous work. Further investigations are needed to unravel in more detail the mechanisms leading to the observed differences between different segmentation pipelines.

Furthermore, the analysis of agreement between the software packages and manual outlines revealed important insights into how MS pathological changes may affect the association between thalamus atrophy and cognitive outcome. First, Bland-Altman revealed a proportional bias with a negative trend of differences between virtually all automated segmentation techniques included in this study (excepted CAT12) and manually derived thalamus measurements, proportional to the magnitude of thalamus size. It seems therefore that the algorithms tend to reduce the gap between smaller and larger thalami, which could negatively impact the study conclusions in several ways. For example, type 1 errors could potentially emerge from invalid comparisons between different structures or tissue types. Also, type 2 errors could occur because sensitivity to true group differences might be obscured by inconsistently localized effects. Nevertheless, automated thalamus segmentations yielded larger effect sizes for the separation of CI vs CP MS patients than manually derived volumes. These discrepancies are most likely explained by the higher level of variability present in the manual data (as indicated by the higher SD, especially for the left thalamus) and a worse level of agreement (ICC) between repeated measures. Future algorithmic developments should be directed towards minimizing proportional bias, since this is likely to significantly influence the statistical power of experiments measuring thalamus volumes.

A discernible amount of variability was found in the manual tracing of the thalamus as evidenced by the intra-rater ICC’s (Derakhshan et al., 2010, Fischl et al., 2002, Houtchens et al., 2007). Owing to the complexity of the cerebral anatomy combined with imaging artefacts (partial volume, intensity inhomogeneity, noise, etc.) present in MRI data, manual outlining is difficult, labor-intensive and time consuming. This particularly applies to the thalamus, which is an agglomeration of smaller nuclei, which leads to an ill-defined boundary of the overall thalamus on conventional MRI, especially in the presence of neurodegeneration. In order to minimize error and reduce variability, we decided to solicit a single expert reader trained in manual tracing on MRI to obtain the highest quality thalamus outlines possible. We did not limit the number of patients or slices and decided to generate thalamus segmentations on each slice, which increases the relevance of this study. Importantly, by using this dataset we were able to objectively compare some of the most widely applied automated segmentation techniques in a multi-center setting, considering the sampling from a large cohort of patients, representative of the full range of a typical RRMS population. Moreover, we have created a valuable set of full manual thalamus outlines of all subjects to provide reference correlations with the cognitive scores.

### 4.1 Limitations

Our study has several limitations, including the absence of a neuropsychological evaluation of the HCs, as well as the assessment of thalamus damage only, which did not allow us to investigate other patterns of microstructural tissue and (deep) GM damage that likely contribute to CI (Damjanovic et al., 2017, Preziosa et al., 2016, Schoonheim et al., 2015, Schoonheim et al., 2012). The choice of the thalamus as a region of interest was motivated by the abundance of literature showing a relationship between damage to the thalamus and cognitive dysfunction in MS patients. As a result, we cannot rule out the possibility that other patterns of more diffuse pathological processes contributed to CI in our MS patients, and a multi-structure imaging and measurement approach is likely needed (Damjanovic et al., 2017, Sastre-Garriga et al., 2020). Concerning image acquisition, (near)isotropic 3D T1-weighted images with similar acquisition parameters were used to obtain thalamus atrophy. In this work we addressed the potential effect of between-center heterogeneity in MRI acquisition in the regression analyses, however, remaining differences between scanners can systematically affect the robustness of the association between deep GM atrophy measurements and cognition across methods (Amiri et al., 2018). A more detailed evaluation of the interaction between MRI acquisition parameters and different thalamus segmentation methods (i.e., the robustness of the various segmentation methods with regards to MRI acquisition parameters) transcended the scope of this study, but should be addressed in future work.

### 4.2 Conclusion

This multi-center study helps to shed light on some previously reported differences between various automated segmentation techniques and how these might influence the relationship between thalamus volume measurements and cognition in MS. It supports the notion that thalamus atrophy is associated with a worse cognitive profile in MS patients. However, one should be cautious when interpreting these findings given the proportional biases that might be present in automated volumetry, especially in smaller and larger thalami, as well as the impact of differences in scanners and acquisition protocols. The approaches work in a multi-center setting, but statistical power is increased by appropriate matching of algorithms with optimal scanners and MRI acquisition parameters. Further research is needed to account for these potential sources of error and ensure the accuracy of these methods in the real-world clinical evaluation of MS patients.
