# Supplementary material for: Manual and automated tissue segmentation confirm the impact of thalamus atrophy on cognition in multiple sclerosis: A multicenter study
Source: Neuroimage Clin. 2020 Dec 25;29:102549. doi: 10.1016/j.nicl.2020.102549 (PMC7787946; doi:10.1016/j.nicl.2020.102549)
Supplement: Supplementary data 1 [file mmc1.pdf]

File S1: A protocol for the manual segmentation of the thalamus, caudate and putamen using high resolution MRI

J. Burggraaff[1*], Y. Liu[2*], J. Simoes[2*], M.P. Wattjes[2], F. Barkhof[2], C.R.G. Guttmann[3], H. Vrenken[2]

*Equally contributing authors

Affiliations
[1] *Department of Neurology, VU University Medical Center (Amsterdam, NL)*
[2]*Radiology and Nuclear Medicine, VU University Medical Center (Amsterdam, NL)*
[3]*Center for Neurological Imaging, Department of radiology, Brigham and Women's Hospital, Harvard Medical School (Boston, MA, USA)*

This segmentation protocol describes how to manually trace, on 3D T1-weighted MRI-scans, three deep grey matter (DGM) structures: the thalamus, caudate, and putamen.

General introduction: edge tracing and manual segmentation process

The segmentation procedure consists of two phases: first, demarcating the edges of the DGM structures on orthogonal scans, and second, outline and fill the inside of the path you defined, using the "bucket" tool, on axial scans. This is done in a "slice-by-slice" manner, respecting the boundaries you previously defined and the anatomical definitions that are specified for each boundary of each structure as described in the instruction manual below. By demarcating the threshold boundaries first, we hope to reduce the risk of inaccuracy.

Some general rules apply to this work, that you should keep in mind while delineating the structures:

- Before you start using this manual it's important that you have enough background knowledge of the objects of interest and their 3D anatomical position/location in the human brain. We recommend you to use the reference books and papers that are listed at the end of the manual that include images of histopathological specimens, MRI, (stereotactic) anatomy and computational 3D reconstructions. Especially the papers on the thalamus can be really helpful to understand its location and shape, and should be studied first.
- Always start with delineating the putamen before the caudate, making it easier to differentiate between the two (and the nucleus accumbens) later on.
- If there is a MS lesion obscuring the border or (parts of) the structure, try to draw a straight line through it, pretending as if it wasn't there. Excluding lesions will artificially decrease the size of the (affected) structure.
- If the scan is of such bad quality (e.g. movement or artefacts) that the DGM structures can't be distinguished from the background you leave out this particular scan and make note of it.

Edge tracing process on (orthogonal) slices

Always start by identifying and demarcating the edges of the structures, using one of the three (orthogonal) orientations, depending on which border of which structure you are planning to trace. Specifically, for detection of the superior, inferior, medial and lateral edges, the sagittal and coronal scans should be used as a reference; to detect the anterior and posterior edges, it's easier to use the axial scans. In addition, be aware of the anatomical definitions that are specified per border and structure. Next, the manual segmentations (outline and fill the inside of the path using the bucket tool) should be performed on the axial scans, respecting the boundaries that you previously defined.

In order to serve as a reference for the manual segmentation process on the axial slices, search for an orthogonal slice (as specified per structure and border below) that reveals the middle part of the structure, where the intensity of the object of interest and its background differ to a large extent [grey matter (GM) versus white matter (WM)] and where the structure is at its largest size. By starting out with searching for the pixels/voxels in the image with sharp intensity changes, makes it easier and generally feasible to identify the object based on visual appearance only. Then, you outline the structure on the orthogonal slices to create demarcation points that will appear as a dot or a line on the axial scans and serve as threshold boundaries for the actual segmentation process (figure 1.1).

Segmentation process on axial slices

Once the edges of the structures are demarcated on the orthogonal images, the manual outline and filling the inside of the path you defined should be done on the axial scans, always staying within the lines (as we only want to include GM and not WM). This procedure is repeated slice-by-slice, respecting the boundaries you previously defined. Once you encounter a boundary of a structure, which can be identified by the very last slice where you find a demarcating dot or line resulting from the edge tracing process (figure 1.1), you've reached the threshold boundary that should not be crossed. In other words, continue the manual segmentations of the structure, using the axial scans, until you've reached this particular point and never go any higher or lower than that. Leave the dot or line (the edge) unchanged, unless GM tissue can still be identified, as shown in figure 1.2.

Figure 1.1 Superior edge of the thalamus, identified as the vertical line on the axial image

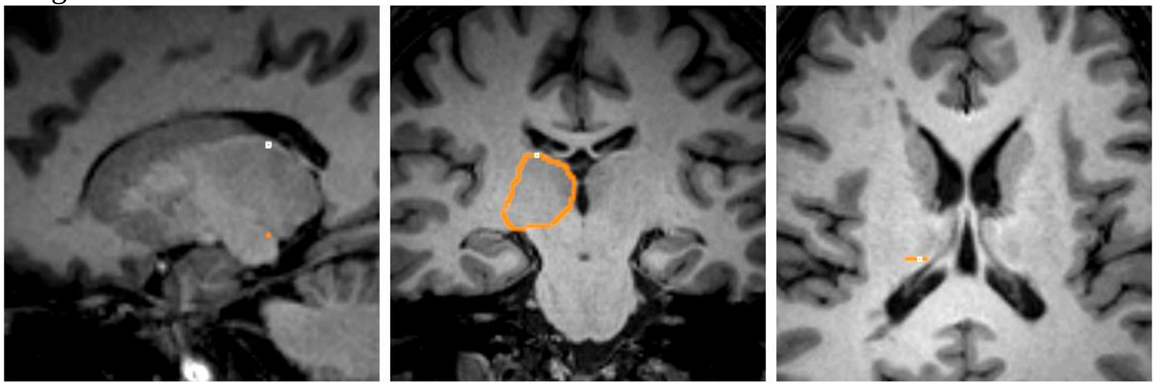

Figure 1.2 Superior edge of the thalamus: GM of the thalamus can still be distinguished

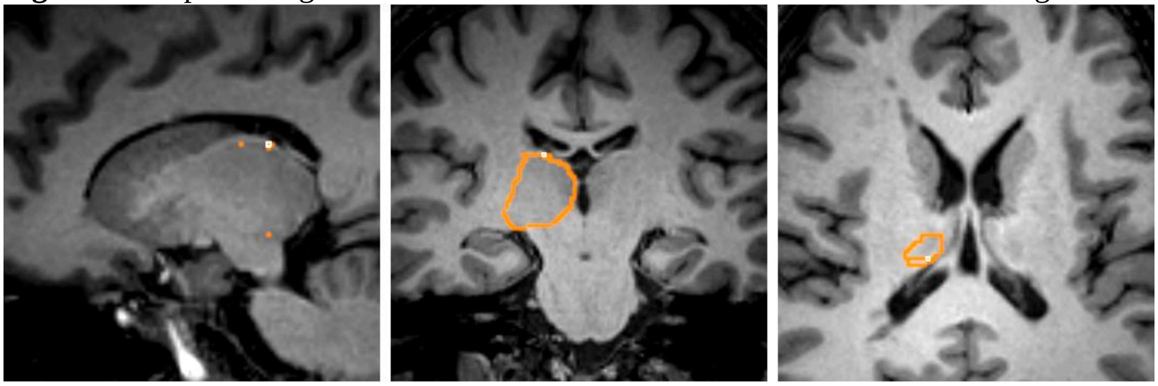

Thalamus

The thalamus is a midline bulb-shaped structure of two halves, situated between the cerebral cortex and the midbrain, symmetrically on each side of the third ventricle. It comprises a system of lamellae (made up of myelinated WM fibers) separating different thalamic subparts, making it difficult to distinguish the thalamus from the background because of the mixed WM-GM voxel intensities. The medial surface of the thalamus constitutes the upper part of the lateral wall of the third ventricle, and is connected to the corresponding surface of the opposite thalamus by a flattened gray band, the interthalamic adhesion (mass intermedia, middle commissure, gray commissure).

<u>Anterior & posterior border: edge tracing process:</u>
To identify the anterior and posterior edges of the thalamus, use the axial plane as a reference. Then, search for a scan revealing the middle part of the structure, where GM can clearly be distinguished from WM and where the structure is at its largest size. Then, outline the thalamus on this particular slice (figure 2.1 and 2.2), to serve as a threshold boundary for the segmentation process on the axial scans.

Figure 2.1 Anterior border of the thalamus

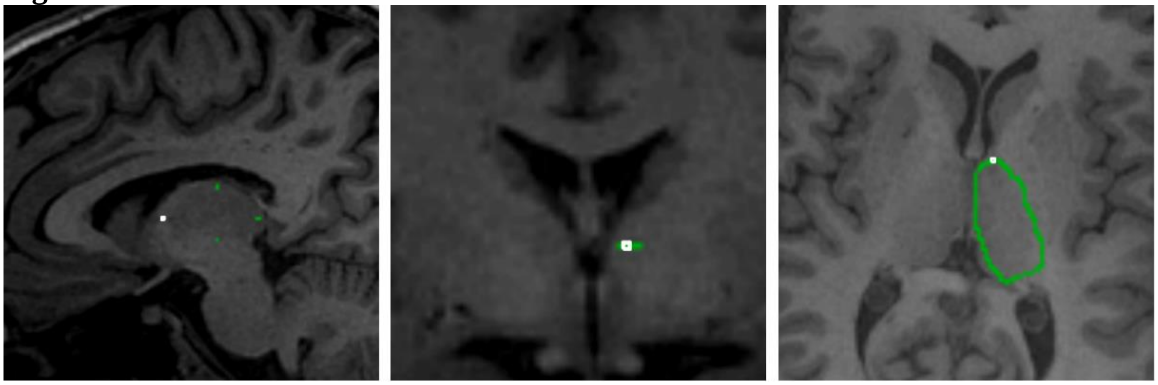

Figure 2.2 Posterior border of the thalamus

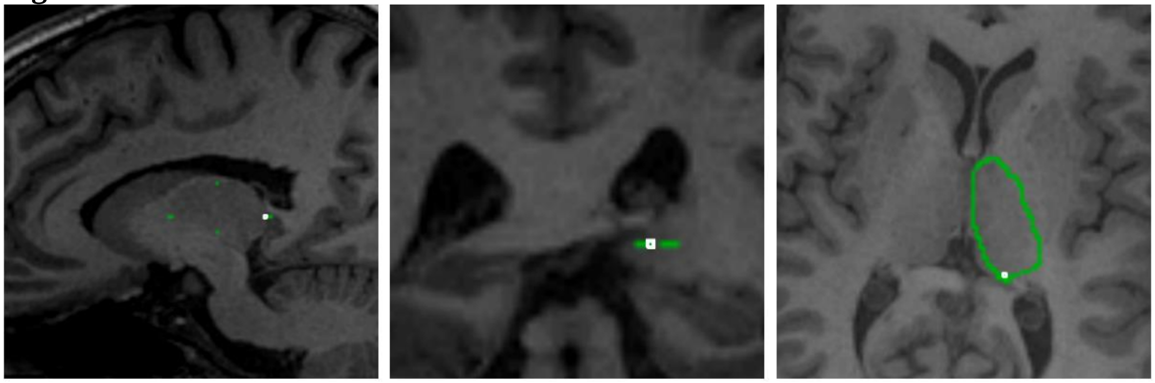

<u>Anterior border: anatomical definition:</u>
Using the anterior commissure (figure 2.3) as a starting point, the thalamus can be identified just posterior to the commissure's clearest coronal view.

Figure 2.3 Anterior commissure indicated by the red arrows

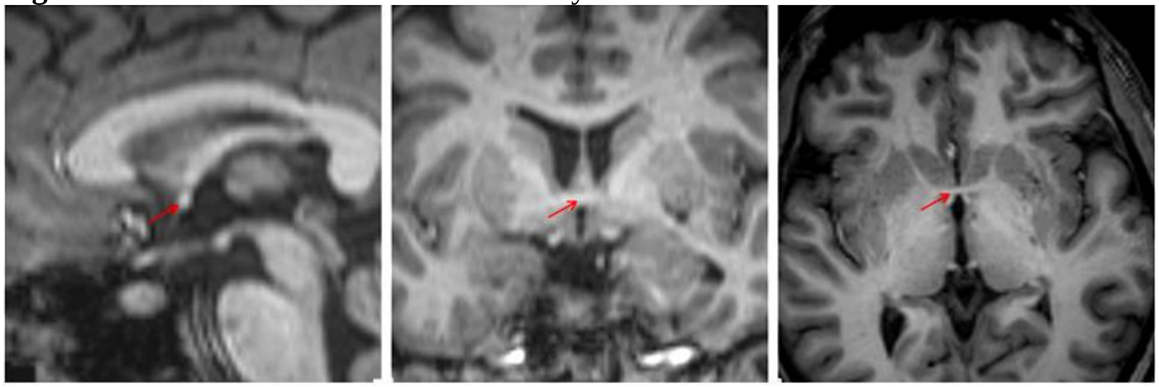

<u>Posterior border: anatomical definition:</u>
Projecting beyond both the superior and inferior colliculi (figure 2.4), the thalamus protrudes to the posterior end until either touching the atrium of the lateral ventricle or the tail of the hippocampus, or both structures. This is most clearly visible in the sagittal plane.

Figure 2.4 Posterior border of the thalamus

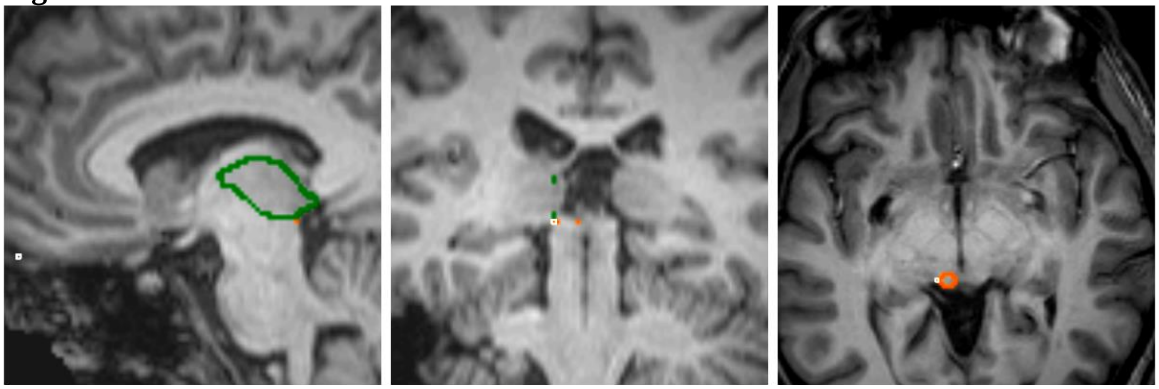

In orange: superior colliculi (right); In green: the thalamus (sagittal view)

Inferior & superior border: edge tracing process:

To identify the superior and inferior border of the thalamus, start with the coronal view as a reference, finding a scan of its middle part, where the GM can be clearly distinguished from the WM and where the thalamus is at its largest size. As the thalamus starts small and ends wider in the coronal view, because of the geniculate bodies that protrude to posterior, we recommend you to define the edges on 2 separate slices: one more anteriorly, where the geniculate bodies are no longer visible, and one more posteriorly, where the geniculate bodies can be distinguished. Then, outline the thalamus on these particular slices (figure 2.5 to 2.6.3), to serve as a threshold boundary for the segmentation process on the axial scans.

One should be cautious when outlining the inferior border of the thalamus, as the more inferior you get, the more difficult it is to differentiate between GM and WM tissue (especially more posteriorly). Most importantly, the lateral geniculate nuclei (LGN) and medial geniculate nuclei (MGN) should be included, as they form part of extensions of the thalamus itself. In the coronal plane, both the LG and the MG are bounded at the inferior border by the CSF superior to the hippocampus and the ambient cistern. (figure 2.6.2 and 2.6.3). Be aware that the LGN and MGN may appear as independent / separate GM areas, intermittent with WM, as they protrude to posterior. However, they should be outlined as one structure, including the WM that is located in between, as shown in figure 2.6.3.

Figure 2.5 Superior border of the thalamus more posteriorly, including the geniculate bodies

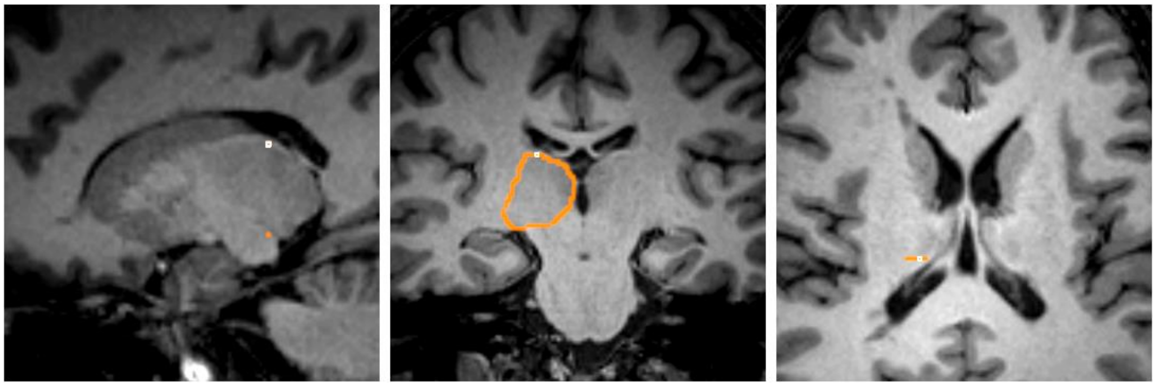

Figure 2.6.1 Inferior border of the thalamus more anteriorly: excluding the geniculate nuclei

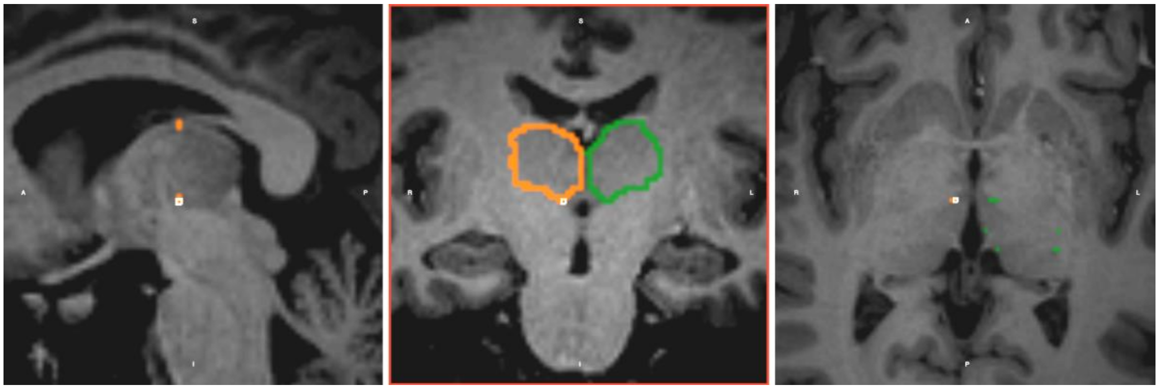

Figure 2.6.2 Inferior border of the thalamus more posteriorly, including the geniculate bodies

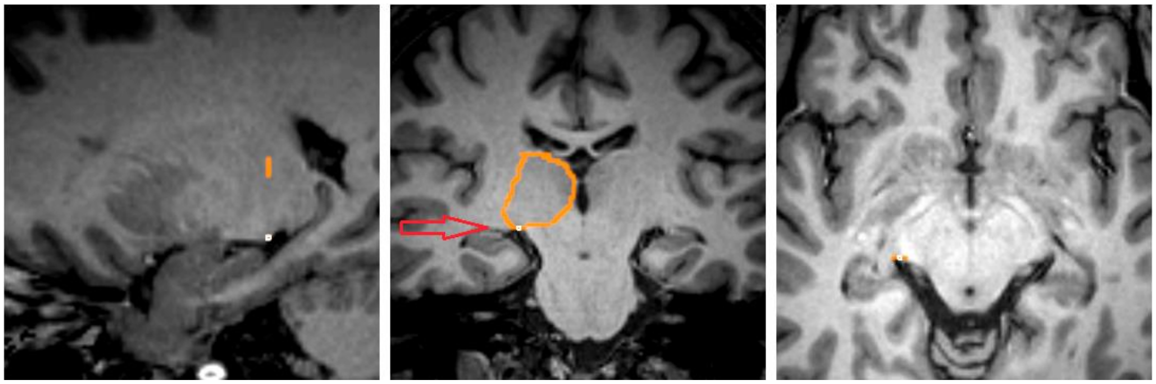

The red arrow indicates the CSF superior to the hippocampus and the ambient cistern

Figure 2.6.3 Inferior border of the thalamus, including the geniculate bodies

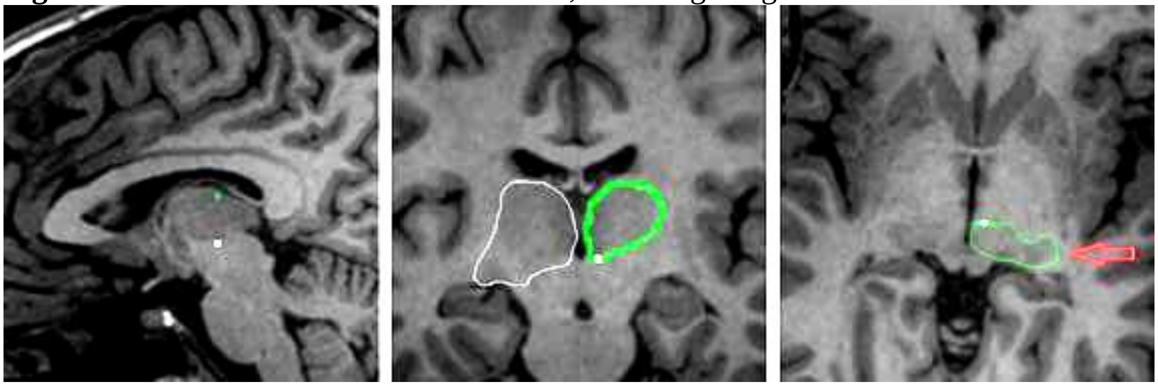

Middle image (coronal view): thick green line indicates the manual outline, excluding the LGN (wrong); in purple you see the outline of the thalamus, including the LGN (correct). Right image (axial view): the green line indicates the thalamus, including the LGN.

Superior border: anatomical definition:

The superior border of the thalamus is bounded by the lateral ventricles. As one moves from anterior to posterior, the thalamus loses its magnitude in the Y plane (anterior-posterior). This causes the coronal height of the thalamus to decrease, thus allowing the fornix to serve as the superior boundary in more posterior slices.

Inferior border: anatomical definition:

The zona incerta (figure 2.7) and its junction with the internal capsule serve as the inferior border of the thalamus, thus excluding the subthalamic nucleus, the substantia nigra and the nucleus rubor (figure 2.8).

More posteriorly, both the LGN and the MGN are bounded at the inferior border by the CSF superior to the hippocampus and the ambient cistern. (figure 2.5, 2.6.2 and 2.6.3)

Figure 2.7 The zona incerta serves as the inferior border of the thalamus

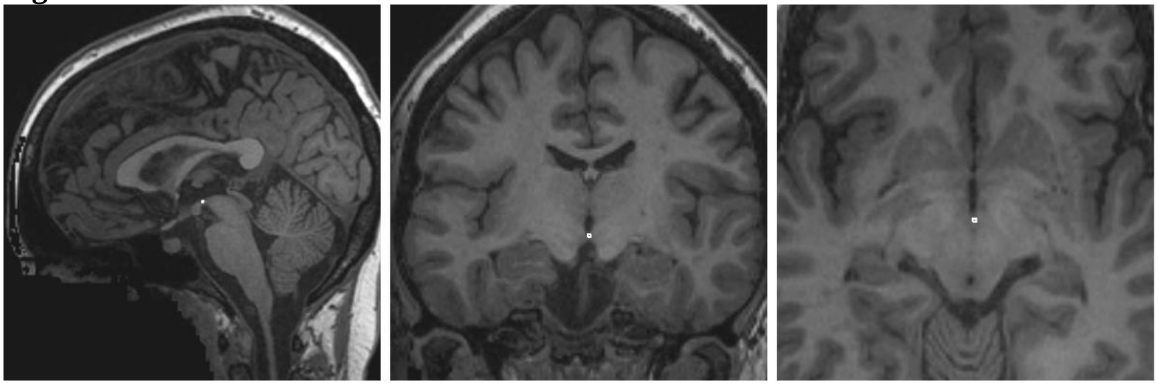

Figure 2.8 The nucleus rubor should be excluded from the thalamus

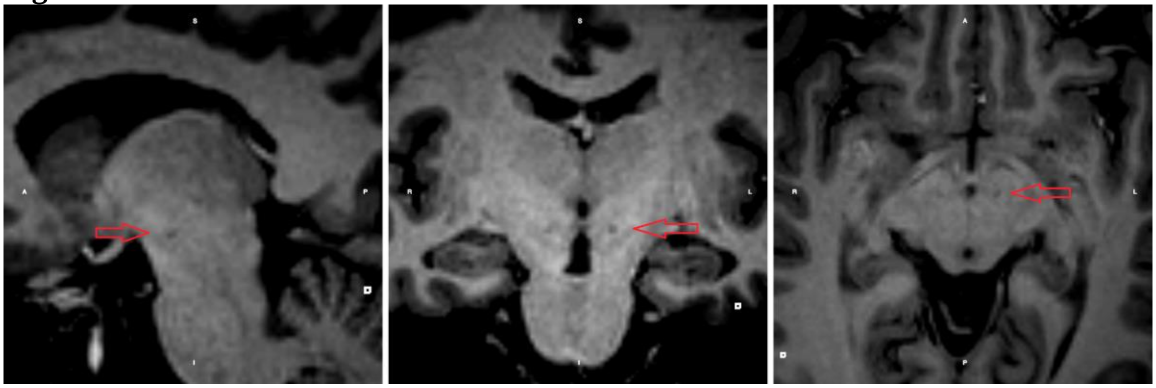

<u>Medial & lateral border: edge tracing process:</u>

To define the medial and lateral border of the thalamus, you can use the same edge tracing delineations as for the superior and inferior border, starting out with the coronal view as a reference (using the same scan as for the inferior and superior borders), finding a scan of its middle part, where the GM can be clearly distinguished from the WM and where the thalamus is at its largest size. As the thalamus starts small and ends wider in the coronal view, because of the geniculate bodies that protrude to posterior, we recommend you to define the edges on 2 separate slices: one more anteriorly, where the geniculate bodies are no longer visible, and one more posteriorly, where the geniculate bodies can be distinguished. Then, outline the thalamus on these particular slices (figures 2.5 to 2.6.3 and), to serve as a threshold boundary for the segmentation process on the axial scans.

<u>Medial border: anatomical definition:</u>

The CSF of the third ventricle serves as the medial border of the thalamus.

Where applicable, portions of the fornix may also serve as a medial border. It is important to note that left and right portions of the thalamus should be traced individually, including the massa intermedia (interthalamic adhesion) when present (figure 2.10).

Figure 2.9 The interthalamic adhesion is indicated by the crosser

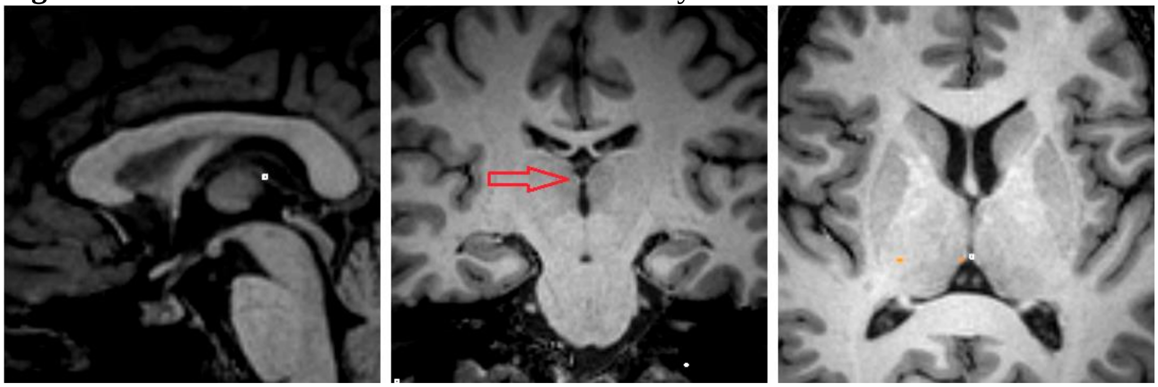

<u>Lateral border: anatomical definition:</u>

Throughout its course in the brain, the thalamus maintains its relationship with the internal capsule as far as its most posterior slices. The medial border of both the genu and the posterior limb of the internal capsule serve as the lateral border of the thalamus, thus separating the thalamus from the adjacent Reticular nucelus.

Reticular nucleus

The reticular nucleus wraps around most of the lateral and rostral thalamus, and by its very distinct and unique anatomical and functional properties,
is considered separate from the rest of the thalamus. It is irregular in shape, varying from a very thin sheet of cells along its lateral route, between lateral nuclei and the internal capsule, and broadening at caudoventral and rostral ends of the thalamus. The cells vary little in size but vary in density along the nucleus, with the lowest density in its lateral, thinnest part.

Care should be given to exclude the three other major subdivisions of the diencephalon: the epithalamus, the hypothalamus and the subthalamus.

Putamen

The putamen is a subdivision of the lenticular nucleus(the other division is the pallidum). From the axial view the lenticular nucleus resembles a rounded triangle (with a lense-like shape) that is divided into two major sections. The putamen forms the lateral part of this triangle. The putamen starts small and ends small in the coronal view. The putamen quickly grows to its greatest size in the middle and in the medial posterior portion it closely resembles a goldfish shape (coronal view). The putamen lies lateral and partially anterior to the thalamus. It is bordered laterally, superiorly, and inferiorly by white matter, making it easy to distinguish the structure from it's background.

<u>Anterior & posterior border: edge tracing process</u>

To identify the anterior and posterior border of the putamen, use the axial view as a reference. Find a scan of its middle part, where the GM is clearly distinguished from the WM and where the putamen is at its largest size. Then, outline the putamen on this

particular slice (figure 3.1 and 3.2), to serve as a threshold boundary for the segmentation process on the axial scans.

Figure 3.1 Anterior border of the putamen

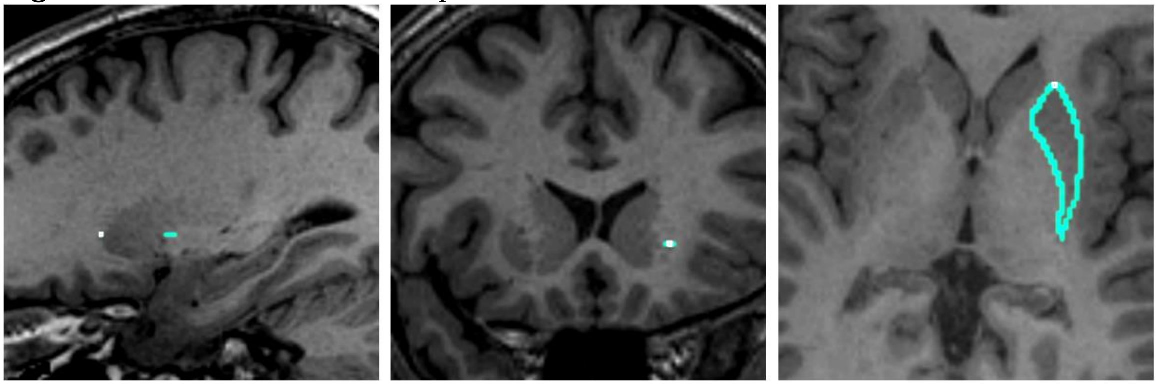

Figure 3.2 Posterior border of the putamen

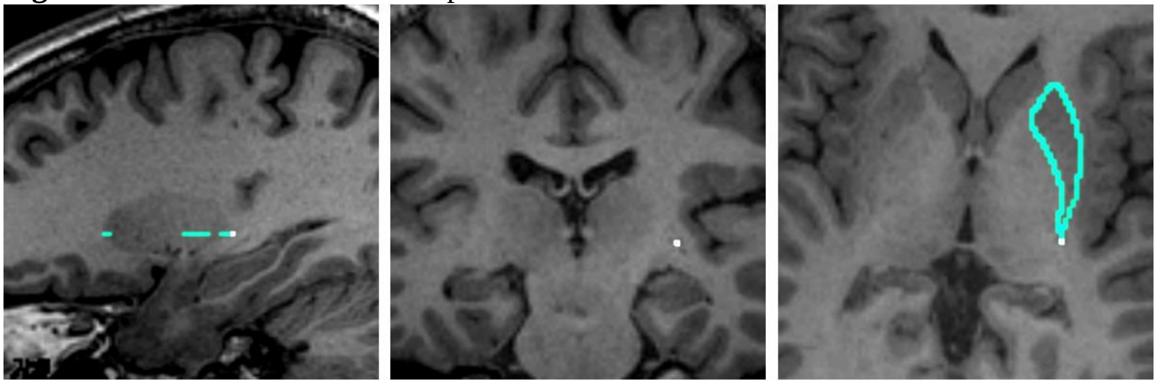

<u>Anterior border: anatomical definition:</u>

In the axial view, the putamen is bounded by the anterior limb of the internal capsule and later as part of the anterior perforated substance. Care should be taken to exclude the perforated substance, closest in appearance to the ventral putamen tissue.

<u>Posterior border: anatomical definition:</u>

Dorsally, the putamen is well-separated from the caudate by parts of the anterior and posterior limb of the internal capsule. Any "projections" or GM “bridges" between the caudate and putamen should be included as part of the caudate. These bridges are thin structures that are obliquely orientated with respect to the anatomical plane.

<u>Inferior & superior border: edge tracing process:</u>

To identify the inferior and superior border of the putamen, start with the sagittal view as a reference, finding a scan of its middle part, where the GM can be clearly distinguished from the WM and where the putamen is at its largest size. Then, outline the putamen on this particular slice (figure 3.3 and 3.4), to serve as a threshold boundary for the segmentation process on the axial scans. Caution should be taken when outlining the inferior part of the putamen, as perivascular spaces (Virchow Robin spaced) should be included (figure 3.5).

Figure 3.3 Superior border of the putamen

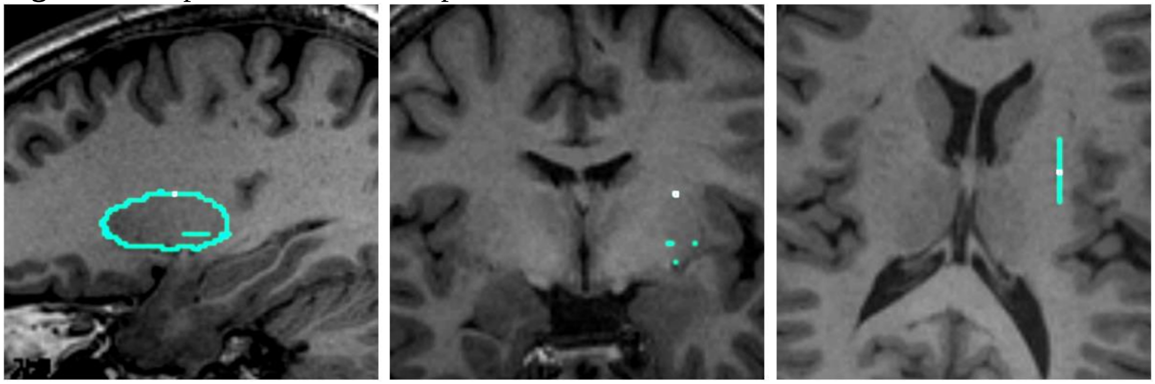

Figure 3.4 Inferior border of the putamen

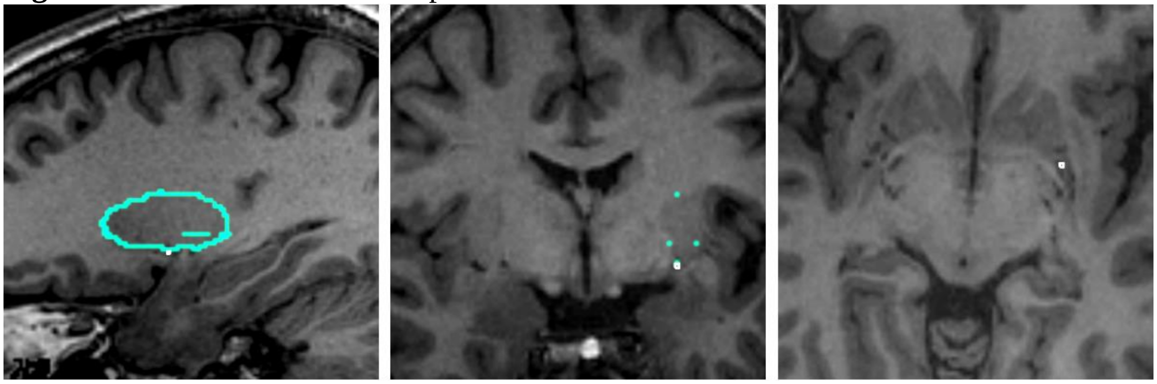

Figure 3.5 Perivascular spaces in the putamen (indicated by the red arrows)

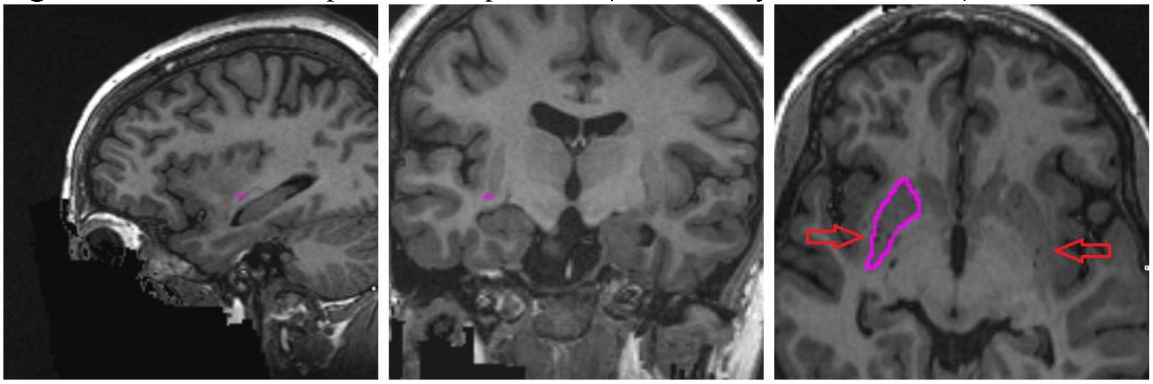

<u>Inferior & Superior border: anatomical definition:</u>

In the coronal view, the Putamen is initially bounded laterally and on its inferior border by the external capsule, and medially and on its superior border by the internal capsule.

<u>Medial & lateral border: edge tracing process:</u>

To identify the medial and lateral border of the putamen, start with the axial view as a reference, finding a scan of its middle part, where the GM can be clearly distinguished from the WM and where the thalamus is at its largest size. Then, outline the putamen on this particular slice (figure 3.6 and 3.7), to serve as a threshold boundary for the segmentation process on the axial scans.

Figure 3.6 Lateral border of the putamen

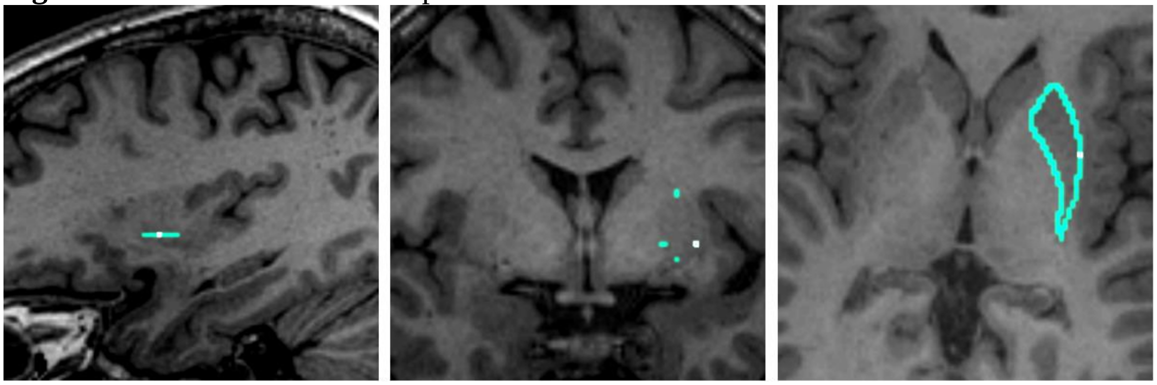

Figure 3.7 Medial border of the putamen

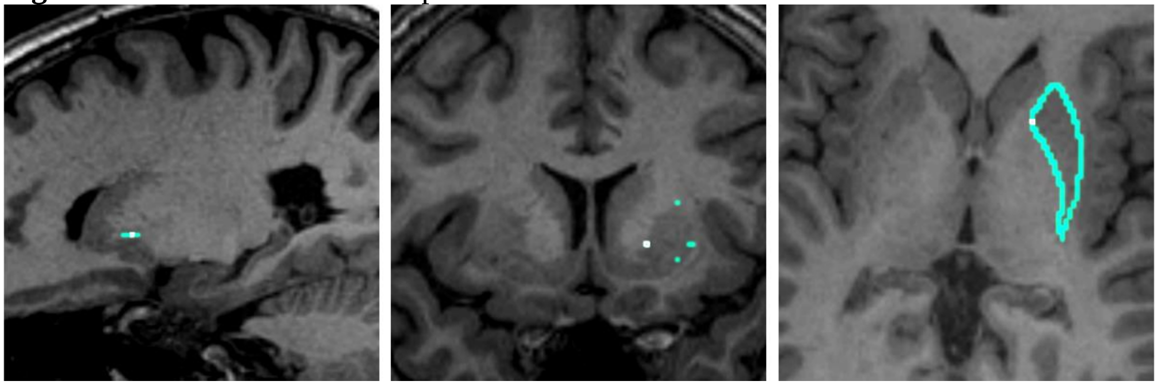

Medial border: anatomical definition:

The external medullary lamina of the globus pallidus serves as delineation of the medial border of the putamen. Further on, the putamen is bordered medially by the pallidum. When the pallidum is not yet present or has already disappeared, the medial border of the putamen is composed of the internal capsule.

Lateral border: anatomical definition:

As a small amount of WM separates the lateral aspect of the putamen from the claustrum, it is important to recognize this structure by its presence and shape, in order to distinguish it from the putamen (figure 3.8.1 and 3.8.2).

Figure 3.8.1 Putamen and claustrum (as indicated by the crosser)

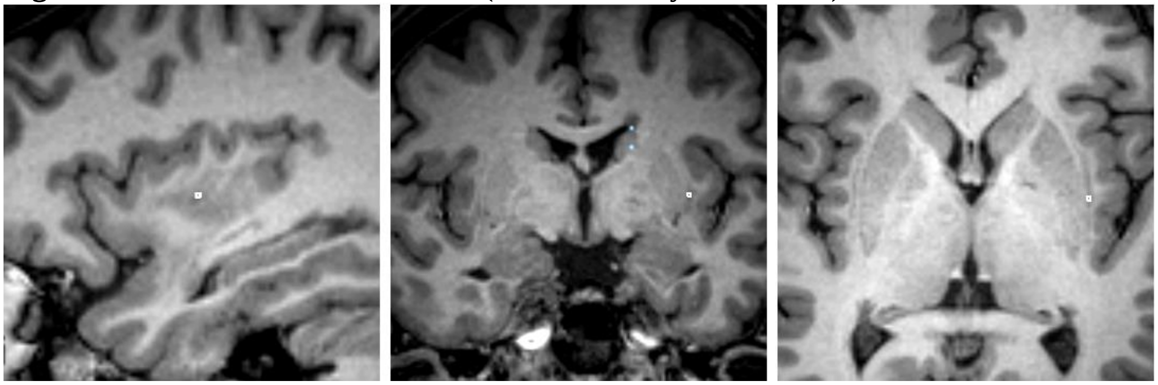

Figure 3.8.2 Putamen (in green) and claustrum (in orange), as indicated by the arrow

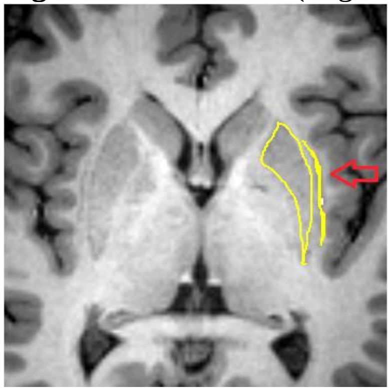

Caudate

The caudate nucleus is one of three basic structures that make up the basal ganglia. The caudate nucleus is an elongated, arched grey mass related throughout its extent to the surface of the lateral ventricle. It is located near the center of the brain, sitting aside the thalamus. There is a caudate nucleus within each hemisphere of the brain. Individually, they resemble a C-shape structure with a wider "head" at the front, tapering to a "body" and a "tail".

Before you start delineating the Caudate, it’s important to differentiate between the caudate, putamen and the nucleus accumbens (Acc) first, which will be explained in further detail below:

First of all, we recommend you to segment the putamen first, using the instructions described earlier. This way, it will be easier to differentiate between the putamen and the other 2 structures later on.

The Nucleus Accumbens

The Acc is located underneath the anterior limb of the internal capsule, between the head of the caudate nucleus and the putamen, parallel to the midline, mostly ahead and inferior to the anterior commissure (ventral to the anterior commissure). The Acc progresses from a round shape to a dorsal-lateral flattened shape. The human Acc is a structure whose

limits are very difficult to define, particularly its anterior limit, which is often indistinguishable from the caudate and the putamen nuclei (dorsal striatum).

To differentiate between the caudate and the Acc, demarcate the following anatomical landmarks that can be identified when moving from posterior to anterior in the coronal plane:

- Anterior limit: The ACC anterior limit has been considered to be the point where the rostral limit of the internal capsule starts separating the caudate from the putamen. As indicated in figure 4.1.

Figure 4.1.1 Anterior limit of the Acc on the right side: internal capsule separates caudate from putamen (as indicated by the red arrow)

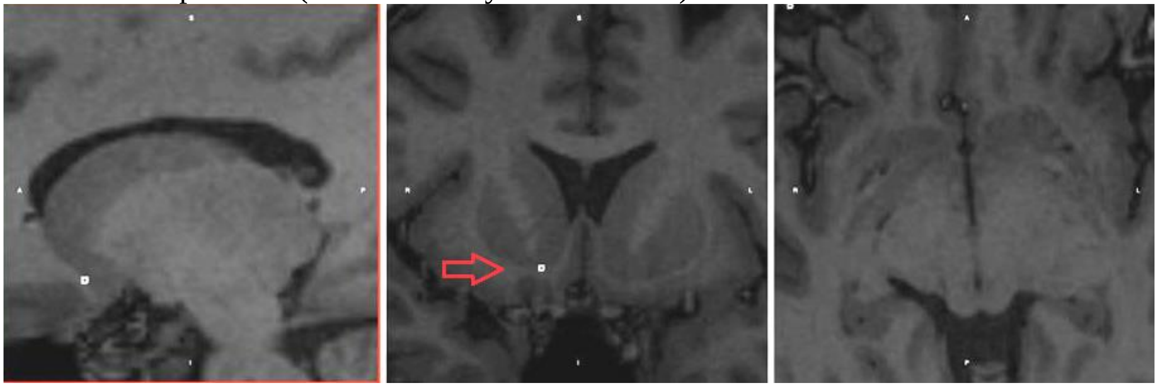

- Posterior limit: In the majority of the atlases, the level of the anterior commissure is accepted as the Acc posterior limit. The Acc ends at the level where the anterior commissure becomes discontinuous, in the coronal plane. As indicated below in figure 4.1.2.

Figure 4.1.2 Posterior limit of the Acc: anterior commissure (as indicated by the red arrow)

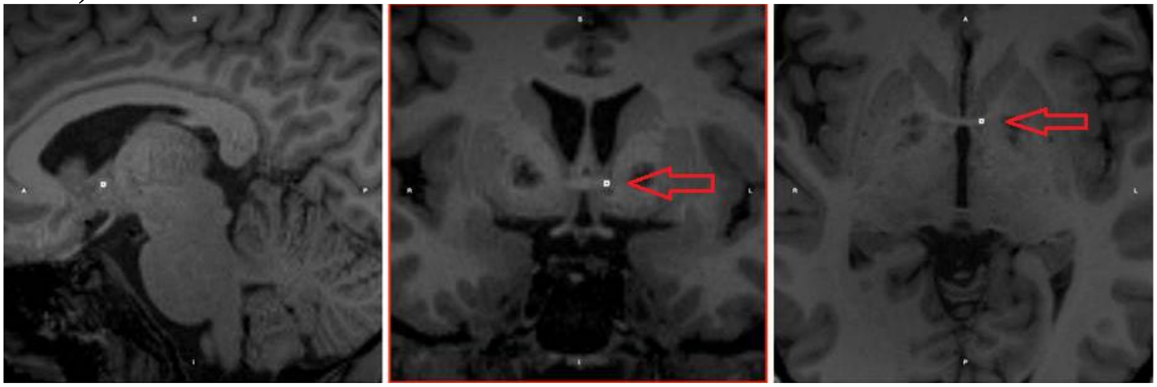

As one moves from posterior to anterior in the coronal plane, we assume that any slice between these 2 demarcation landmarks, includes GM that forms either part of the Acc or the caudate. At the level of the brain where the Acc and caudate are both included (the area in between the anatomical landmarks), you use the bottom of the lateral ventricles, as the inferior border of the caudate, drawing a straight vertical line at that level (figure

4.1.3 and 4.1.4: left caudate). Any GM inferior to that level (bottom ventricles) is probably (part of) the Acc and should not be included as part of the delineation of the caudate. At the level of the brain where you have encountered the anterior limitation landmark, any GM that lies ventral to this demarcation point should be outlined as part of the (head of) the caudate (figure 4.1.3 and 4.1.4: right caudate).

Figure 4.1.3 Anteior limit of the Acc: delineation of the right caudate (at the point of anterior limit) and the left caudate (dorsal to the anterior limit), using the bottom of the ventricles as the inferior border

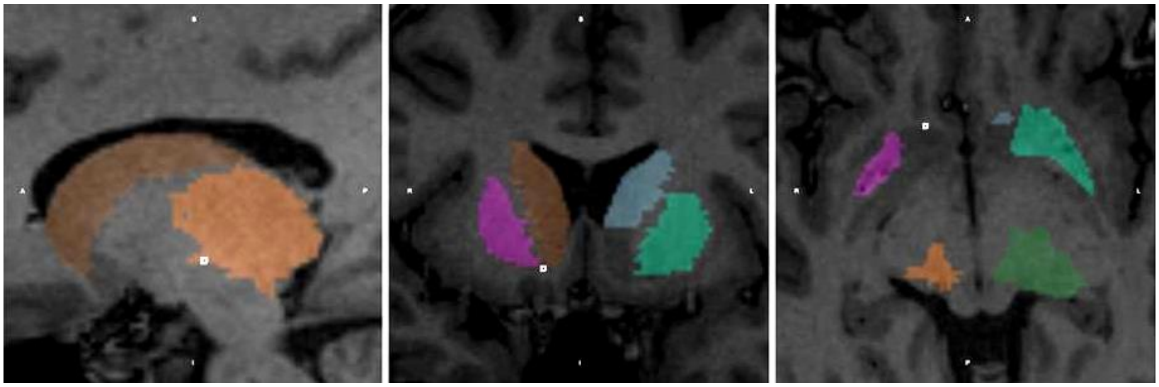

In brown: right caudate; in orange: right thalamus; in pink: right putamen; in blue: left caudate; in green: left thalamus; in turquoise: left putamen.

Because the bottom of the ventricles will not follow a perfect horizontal line when moving from posterior to anterior (as you can see in the sagittal plane), images may appear as intermitted delineations on the axial images, which should be corrected for by filling the rest of the structure (this will fill up the corner that you see on the sagittal plane in figure 4.1.4):

Figure 4.1.4 Intermitted delineations in the axial plane should be corrected for

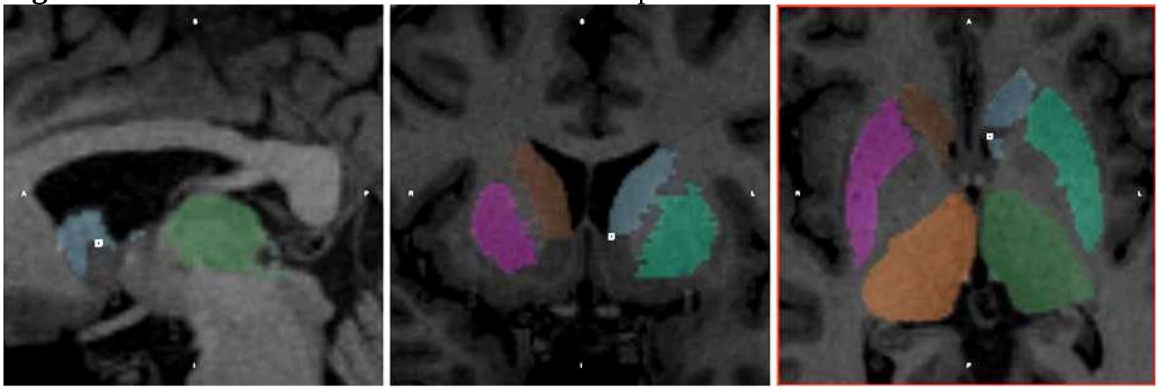

In brown: right caudate; in orange: right thalamus; in pink: right putamen; in blue: left caudate; in green: left thalamus; in turquoise: left putamen.

The delineations of the head of the caudate ventral to the anterior limits, will appear on the axial images as depicted in figure 4.1.5, and should not be corrected for as the dorsal aspect is probably part of the Acc.

Figure 4.1.5 Delineations of the caudate ventral to the anterior limits:

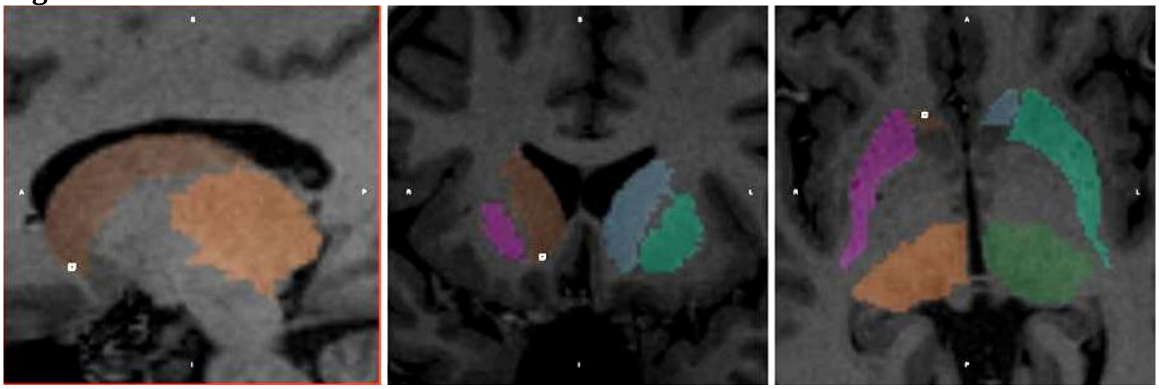

In brown: right caudate; in orange: right thalamus; in pink: right putamen; in blue: left caudate; in green: left thalamus; in turquoise: left putamen.

<u>Anterior & posterior border: edge tracing process:</u>

To identify the anterior and posterior border of the caudate, use the sagittal view as a reference. Find a scan of its middle part, where the GM is clearly distinguished from the WM and where the caudate is at its largest size. Then, outline the caudate on this particular slice (figure 4.2 and 4.3), to serve as a threshold boundary for the segmentation process on the axial scans. Caution should be taken when outlining the tail of the caudate, as it is separated from the head of the caudate on the axial images, however, should be included in the segmentation process (figure 4.4).

Figure 4.2 Anterior border of the caudate

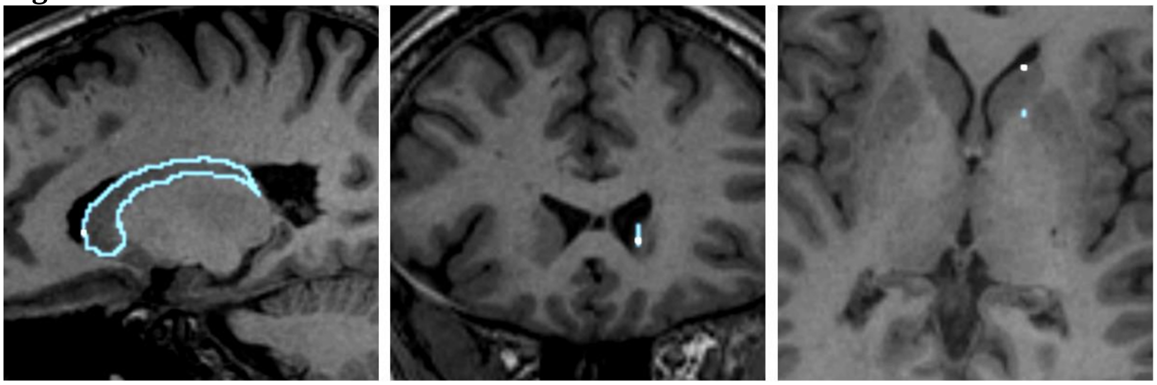

Figure 4.3 Posterior border of the caudate

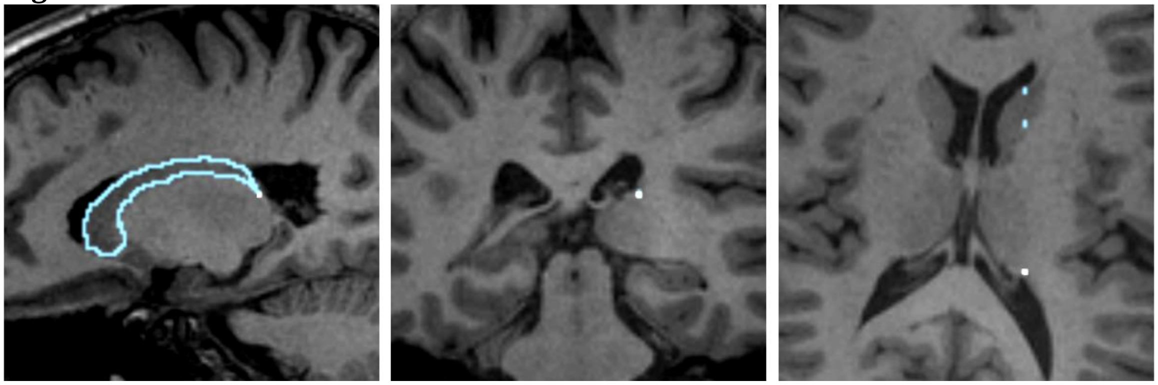

Figure 4.4 Tail of the caudate

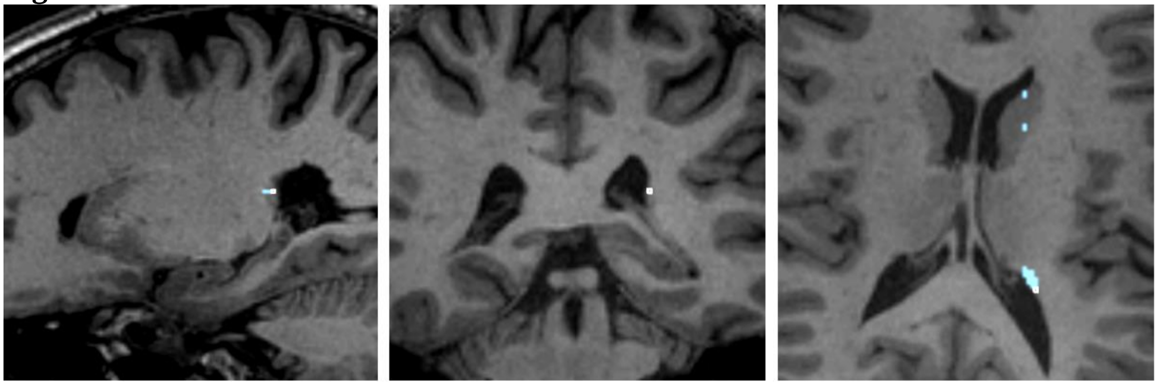

Anterior border: anatomical definition:

Its enlarged anterior portion, or head, lies rostral to the thalamus and bulges into the anterior horn of the ventricle. The head of the caudate nucleus and the putamen are separated by fibers of the anterior limb of the internal capsule, except rostroventrally where continuity is maintained. As described earlier, any "projections" or tissue "bridges" between the caudate and putamen should be included as part of the caudate.

Posterior border: anatomical definition:

The dorsal aspect of the caudate, surrounded by WM, has no adjoining structures that may interfere with its identification.

It should be decided that the tail of the caudate terminates at a point where it is not clearly visible anymore (figure 4.3), as opposed to some sort of cutoff point that is determined by the appearance of another structure.

Inferior & superior border: edge tracing process:

Inferior border:

Beginning rostrally and ending caudally, the following guidelines apply to the tracing of the inferior edges of the caudate: at the level of the brain, occupied by the Acc, the inferior border of the caudate is identified as described earlier (see the part on: differentiation between the caudate and the Acc). Moving further from Acc to more posteriorly, the inferior border can be identified in the same manner as described below for the superior border.

Superior border:

To identify the superior border of the caudate, start with the sagittal view as a reference (using the same scan as for the anterior and posterior borders) finding a scan of its middle part, where the GM can be clearly distinguished from the WM and where the putamen is at its largest size. Then, outline the caudate on this particular slice (figure 4.5, to serve as a threshold boundary for the segmentation process on the axial scans.

Figure 4.5 Superior border of the caudate

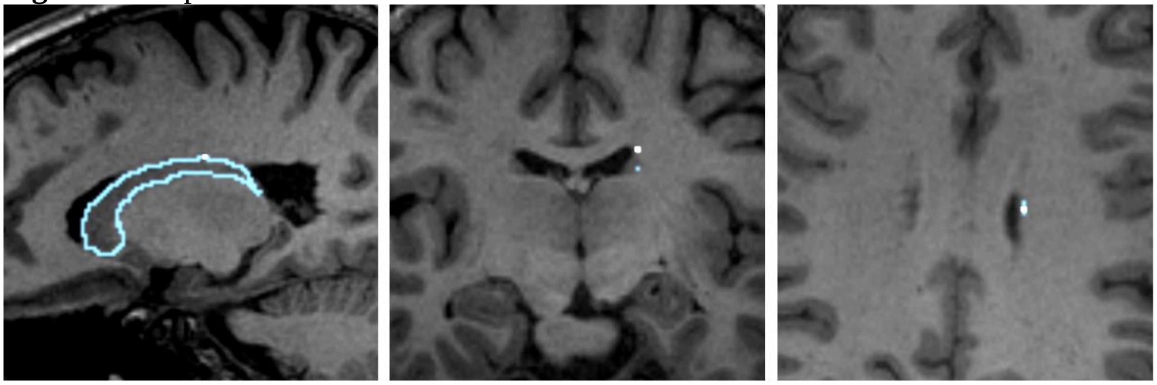

Inferior & superior border: anatomical definitions:

The inferior boundary of the caudate starts directly above the Acc, at the level of the anterior horn of lateral ventricle or subventricular zone. The caudate superior boundary lies above the thalamus and lateral to the confluence of the anterior and posterior horns of the lateral ventricles.

Medial & lateral border: edge tracing process:

To identify the medial and lateral border of the caudate, start with the axial view as a reference, finding a scan of its middle part, where the GM can be clearly distinguished from the WM and where the caudate is at its largest size. Then, outline the caudate on this particular slice (figure 4.5 and 4.6), to serve as a threshold boundary for the segmentation process on the axial scans.

Figure 4.5 medial border of the caudate

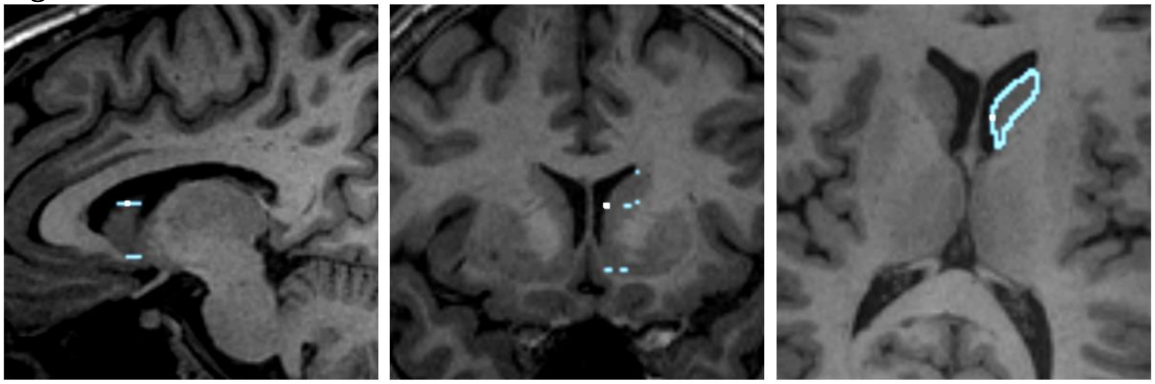

Figure 4.6 Lateral border of the caudate

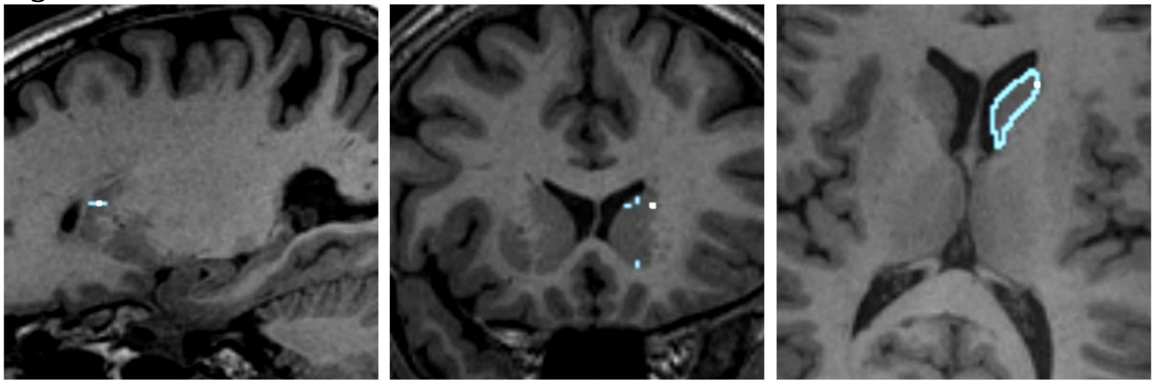

Medial border: anatomical definition:

Care should be given not to include cerebrospinal fluid or meningeal artifacts on the medial aspect of each caudate.

Lateral border: anatomical definition:

The lateral delineation of the caudate (the anterior and further on– posterior limb of the internal capsule) is somewhat easier to identify due to its distinctive light colored appearance (figure 4.7). Lateral projections of caudate tissue (projecting towards and in some cases abutting on the putamen) should be included in the traces.

Figure 4.7 lateral ventricle (pink), caudate (blue) and the internal capsule (red arrow)

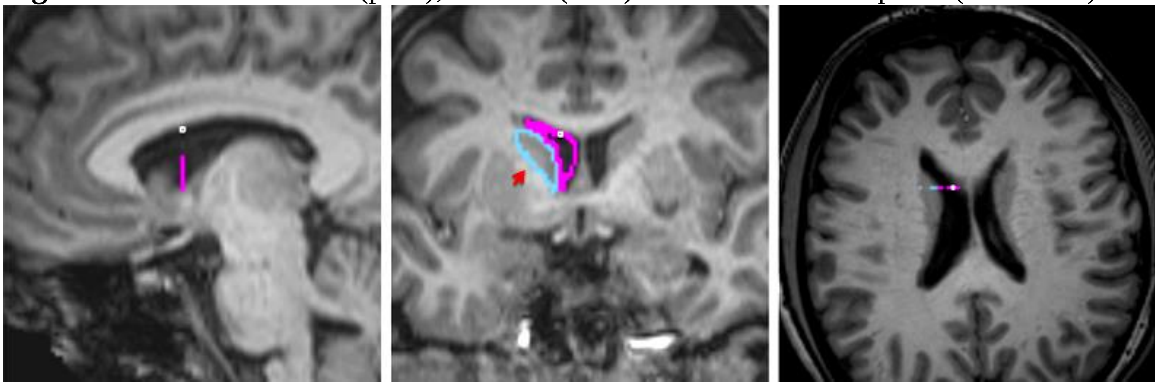

References:

Carpenter, Malcolm B. Core Text of Neuroanatomy. Baltimore: Williams & Wilkins, 1991.

Duvernoy, Henri M. The Human Brain Surface, Three-Dimensional Sectional Anatomy and MRI. New York: Springer-Verlag Wien, 1991.

Gray, Henry. Anatomy, Descriptive and Surgical. Philadelphia: Running Press, 1974.
Kahle, Werner. Color Atlas of Human Anatomy, Nervous System and Sensory Organs. New York: Theme Medical Publishers, 1993.

Martin, John H. Neuroanatomy: Text and Atlas. New York: Elsevier Publishing Co., Inc., 1989.

Focke NK, Trost S Paulus W, Falkai P, Gruber O Do manual and voxel-based morphometry measure the same? A proof of concept study. Front Psychiatry. 2014 Apr 8;5:39. doi: 10.3389/fpsyt.2014.00039

Morel, A., Magnin, M., Jeanmonod, D., 1997. Multiarchitectonic and stereotactic atlas of the human thalamus. J. Comp. Neurol. 387, 588–630.

Yelnik, J., Bardinet, E., Dormont, D., Malandain, G., Ourselin, S., Tande, D., Karachi, C., Ayache, N., Cornu, P., Agid, Y., 2007. A three-dimensional, histological and deformable atlas of the human basal ganglia: I. Atlas construction based on immunohistochemial and MRI data. NeuroImage 34, 618–638.

A. Krauth et al. A mean three-dimensional atlas of the human thalamus: Generation from multiple histological data. NeuroImage 49 (2010) 2053–2062

L. Lucas-Neto et al. Three dimensional anatomy of the human nucleus accumbens. Acta Neurochir (2013) 155:2389–2398

Massey LA, Yousry TA. Anatomy of the substantia nigra and subthalamic nucleus on MR imaging. Neuroimaging Clin N Am. 2010 Feb;20(1):7-27.
